# ﻿Comprehensive treatise of *Hevansia* and three new genera *Jenniferia*, *Parahevansia* and *Polystromomyces* on spiders in Cordycipitaceae from Thailand

**DOI:** 10.3897/mycokeys.91.83091

**Published:** 2022-07-26

**Authors:** Suchada Mongkolsamrit, Wasana Noisripoom, Kanoksri Tasanathai, Noppol Kobmoo, Donnaya Thanakitpipattana, Artit Khonsanit, Booppa Petcharad, Baramee Sakolrak, Winanda Himaman

**Affiliations:** 1 Plant Microbe Interaction Research Team, National Center for Genetic Engineering and Biotechnology (BIOTEC), 113 Thailand Science Park, Phahonyothin Road, Khlong Nueng, Khlong Luang, Pathum Thani, 12120, Thailand Plant Microbe Interaction Research Team, National Center for Genetic Engineering and Biotechnology (BIOTEC) Pathum Thani Thailand; 2 Department of Biotechnology, Faculty of Science and Technology, Thammasat University, Pathum Thani, 12120, Thailand Thammasat University Pathum Thani Thailand; 3 Forest Entomology and Microbiology Research Group, Forest and Plant Conservation Research Office, 61 Department of National Parks, Wildlife and Plant Conservation, Phahonyothin Road, Chatuchak, Bangkok, 10900, Thailand Forest Entomology and Microbiology Research Group, Forest and Plant Conservation Research Office Bangkok Thailand

**Keywords:** Cordycipitaceae, *Hevansia*, *Jenniferia*, *Parahevansia*, *Polystromomyces*, spider pathogenic fungi

## Abstract

Collections of pathogenic fungi found on spiders from Thailand were selected for a detailed taxonomic study. Morphological comparison and phylogenetic analyses of the combined ITS, LSU, *tef1*, *rpb1* and *rpb2* sequence data indicated that these specimens formed new independent lineages within the Cordycipitaceae, containing two new genera occurring on spiders, i.e. *Jenniferia***gen. nov.** and *Polystromomyces***gen. nov.** Two new species in *Jenniferia*, *J.griseocinerea***sp. nov.** and *J.thomisidarum***sp. nov.**, are described. Two strains, NHJ 03510 and BCC 2191, initially named as *Akanthomycescinereus* (*Hevansiacinerea*), were shown to be part of *Jenniferia*. By including sequences of putative *Hevansia* species from GenBank, we also revealed *Parahevansia* as a new genus with the ex-type strain NHJ 666.01 of *Pa.koratensis*, accommodating specimens previously named as *Akanthomyceskoratensis* (*Hevansiakoratensis*). One species of *Polystromomyces*, *Po.araneae***sp. nov.**, is described. We established an asexual-sexual morph connection for *Hevansianovoguineensis* (Cordycipitaceae) with ex-type CBS 610.80 and proposed a new species, *H.minuta***sp. nov.** Based on characteristics of the sexual morph, *Hevansia* and *Polystromomyces* share phenotypic traits by producing stipitate ascoma with fertile terminal heads; however, they differ in the shape and colour of the stipes. Meanwhile, *Jenniferia* produces non-stipitate ascoma with aggregated superficial perithecia forming a cushion. A new morphology of ascospores in *Jenniferia* is described, illustrated and compared with other species in Cordycipitaceae.

## ﻿Introduction

Members of Cordycipitaceae (Hypocreales, Ascomycota) are parasitic on spiders (Araneae) and several orders of insects from larva to adult states (Sung et al. 2007; Shrestha et al. 2016). Several species of this family are recognised for their economic importance, such as *Cordycepsmilitaris* (L.) Fr., a famous traditional Chinese medicine, edible mushroom and source of bioactive compounds (Wu et al. 2021) and others that are being used or developed as biopesticides against different insect pests (Wang et al. 2019; Sun et al. 2020). Seventeen genera are established in this family from combined molecular phylogenetic and morphological evidence (Zare and Gams 2016; Kepler et al. 2017; Mongkolsamrit et al. 2018, 2020b; Thanakitpipattana et al. 2020; Wang et al. 2020; Zhang et al. 2020). Recently, the genera *Pseudogibellula* Samson & H.C. Evans and *Pleurodesmospora* Samson, W. Gams & H.C. Evans were clarified, based on molecular phylogenetic analyses and confirmed to be members of Cordycipitaceae (Chen et al. 2021b; Mongkolsamrit et al. 2021), suggesting that the taxonomic diversity of this family is still under-explored.

Arthropod pathogenic fungi in Cordycipitaceae have a distinctive fleshy texture and pallid (white to yellow) to brightly coloured stipitate stromata with loosely embedded or superficial perithecia. Species with these features include *Cordycepsmilitaris* (L.) Fr., *Blackwellomycespseudomilitaris* (Hywel-Jones & Sivichai) Spatafora & Luangsa-ard, *Flavocilliumbifurcatum* H. Yu, Y.B. Wang, Y. Wang, Q. Fan & Zhu L. Yang and *Samsoniellainthanonensis* Mongkols., Noisrip., Thanakitp., Spatafora & Luangsa-ard (Sung et al. 2007; Kepler et al. 2017; Mongkolsamrit et al. 2018; Wang et al. 2020). Nonetheless, some Cordycipitaceae species are characterised by possessing non-stipitate ascomata, such as *Akanthomycesthailandicus* Mongkols., Spatafora & Luangsa-ard and *Gibellulacebrennini* Tasan., Kuephadungphan & Luangsa-ard (Mongkolsamrit et al. 2018; Kuephadungphan et al. 2020), which are parasitic on the spiders, *Hyperdermiumpulvinatum* J.F. White, R.F. Sullivan, Bills & Hywel-Jones and *H.caulium* (Berk. & M.A. Curtis) P. Chaverri & K.T. Hodge occurring on scale insects (Sullivan et al. 2000; Chaverri et al. 2008).

*Hevansia* and *Gibellula* were separated from other genera, based on monophyletic clades in the Cordycipitaceae (Kepler et al. 2017). *Hevansia* was erected with *H.novoguineensis* (synonym: *Akanthomycesnovoguineensis* Samson & B.L. Brady) as the type species infecting spiders collected from Papua New Guinea (Samson and Brady 1982). *Hevansia* and *Gibellula* species are specialised parasites on spiders that inhabit the undersides of leaves. However, the asexual morph of *Hevansia* differs from *Gibellula* in the production of phialides in monolayer with mono- or polyphialidic conidiogenous cells, whereas species in *Gibellula* produce the primary synnemata bearing predominantly aspergillus-like conidiophores or occasionally growing penicillate or granulomanus-like conidiophores (Samson and Brady 1982; Samson and Evans 1992; Kuephadungphan et al. 2020, 2022).

At present, most of the species in *Hevansia* have been described, based on asexual morphs that were reported from China, Papua New Guinea, Sri Lanka, Taiwan and Thailand (Samson and Evans 1974; Samson and Brady 1982; Hywel-Jones 1996; Hsieh et al. 1997; Huang et al. 2000). *Hevansianelumboides*, the only species from Japan, has been accepted and described, based on sexual characters producing short stipes with fertile terminal heads, immersed perithecia and ascospores disarticulating into part-spores (Kobayasi and Shimizu 1977; Kepler et al. 2017). The sexual morph of *Gibellula* is well-known for forming a torrubiella-like state and ascospores that disarticulate into part-spores. Species in *Gibellula* have been reported from several countries including China, Ecuador, Ghana, Taiwan and Thailand (Samson and Evans 1992; Hsieh et al. 1997; Kuephadungphan et al. 2020; Chen et al. 2021a).

From surveys of arthropod pathogenic fungi in Thailand’s national parks, collections of pathogens on spiders were found on the underside of leaves from forest plants. Based on the macroscopic features of the sexual morph, some specimens possess non-stipitate ascomata with aggregated superficial perithecia forming a cushion. In contrast, some specimens have stipes with fertile heads at the terminal part arising from the spiders’ abdominal region which closely match with *H.nelumboides*. Asexually reproductive species that produce several synnemata on spiders were also included in this study. The goal of these investigations is to elucidate the phylogenetic and taxonomic placement of these collections of parasitic fungi on spiders through multi-locus molecular phylogenetic analyses and the observation of diagnostic macro- and micro-morphological characteristics. Additionally, this work has allowed us to refine the diagnostic characters of the species classification of *Hevansia*.

## ﻿Materials and methods

### ﻿Specimen collection and isolation

The fungal specimens were collected in forests during the rainy season from 2009 to 2020. The specimens of fungi occurring on spiders found on the underside of living leaves of forest plants were collected carefully to preserve host and fungal structures, then were put in plastic boxes and carried to the laboratory for isolation. The materials were examined under a stereomicroscope (Olympus SZ61). The protocol for isolation from sexual and asexual morphs followed previous studies (Luangsa-ard et al. 2018; Mongkolsamrit et al. 2018). The cultures were grown on potato dextrose agar (PDA; freshly diced potatoes 200 g, dextrose 20 g, agar 15 g, in 1 litre distilled water) and deposited at the BIOTEC Culture Collection (BCC), Thailand. The specimens were dried in an electric food dryer (50–55 °C) overnight and stored in plastic boxes before storage at the BIOTEC Bangkok Herbarium (BBH), National Biobank of Thailand. The identification of the spider hosts was conducted after cultures of fungal pathogens were acquired. The spider hosts were identified, based on morphological characteristics, such as eyes, cephalic regions and legs (Deeleman-Reinhold 2001).

### ﻿Morphological observation

Important macroscopic and microscopic features of the fungal specimens were observed using a stereomicroscope (Olympus CX31) and a compound microscope (Olympus SZ61). The fungal materials, including perithecia, asci, ascospores, phialides and conidia, were mounted on microscope slides and stained in lactophenol cotton blue solution for observation. The characteristics of these materials (shape and size) were determined and measured according to Mongkolsamrit et al. (2018, 2020b). Cultures were grown on oatmeal agar (OA, Difco, oatmeal 60 g, agar 12.5 g, in 1 litre distilled water) and PDA agar plates at 25 °C under light/dark condition (L:D = 14:10) for 21 days, depending on the sporulation in culture. The colours of the specimens and colonies grown on OA and PDA were described and codified following the Royal Horticultural Society (RHS 2015).

### ﻿DNA extraction, amplification and sequencing

Genomic DNA was extracted from the mycelia of 10–14 days old cultures on PDA using a modified cetyltrimethyl ammonium bromide (CTAB) method as previously described in Mongkolsamrit et al. (2009). Nuclear loci were sequenced, including the nuc rDNA region encompassing the internal transcribed spacers (ITS), ITS1 and ITS2, the partial gene regions of the nuc 28S rDNA (Large Subunit Ribosomal DNA: LSU), the translation elongation factor-1α gene (*tef1*), the largest (*rpb1*) and second largest (*rpb2*) subunits of RNA polymerase II. Polymerase chain reaction (PCR) primers used to amplify these markers were ITS5 and ITS4 for ITS (White et al. 1990), LROR and LR5 for LSU (Vilgalys and Hester 1990; Rehner and Samuels 1994), 983F and 2218R for *tef1* (Rehner and Buckley 2005), CRPB1 and RPB1Cr for *rpb1* (Castlebury et al. 2004), RPB2-5F2 and RPB2-7Cr for *rpb2* (Liu et al. 1999; O’Donnell et al. 2007). The thermocycler conditions for PCR amplifications used in this study followed the method described in Sung et al. (2007). The purified PCR products were sequenced with PCR amplification primers for Sanger dideoxy sequencing. The sequences obtained in this study were deposited in GenBank (Table 1).

**Table 1.** List of taxa included in the phylogenetic analyses and their GenBank accession numbers.

Species	Code	Host/ Substratum	GenBank accession numbers					References
			ITS	LSU	*tef1*	*rpb1*	*rpb2*	
*Akanthomycesaculeatus*	HUA 772	Lepidoptera; Sphingidae				—	—	Sanjuan et al. (2014)
*A.aculeatus*	HUA 186145^T^	—	—			—	—	Kepler et al. (2017)
*A.kanyawimiae*	TBRC 7244^T^	Araneae; spider				—	—	Mongkolsamrit et al. (2018)
*A.lecanii*	CBS 101247	Homoptera	—				—	Sung et al. (2001); Spatafora et al. (2007)
*A.sulphureus*	TBRC 7248^T^	Araneae; spider						Mongkolsamrit et al. (2018)
*A.thailandicus*	TBRC 7245^T^	Araneae; spider		—		—		Mongkolsamrit et al. (2018)
*A.waltergamsii*	TBRC 7252^T^	Araneae; spider						Mongkolsamrit et al. (2018)
*Ascopolyporuspolychrous*	P.C. 546	Plant	—				—	Chaverri et al. (2005)
*A.villosus*	ARSEF 6355	Plant		—			—	Bischoff et al. (2005); Chaverri et al. (2005)
*Beauveriabassiana*	ARSEF 1564^T^	Lepidoptera		—				Rehner et al. (2011)
*B.bassiana*	ARSEF 7518	Hymenoptera		—				Rehner et al. (2011)
*Blackwellomycesaurantiacus*	BCC 85060^T^	Lepidoptera						Mongkolsamrit et al. (2020b)
*B.aurantiacus*	BCC 85061	Lepidoptera						Mongkolsamrit et al. (2020b)
*B.pseudomilitaris*	BCC 1919^T^	Lepidoptera	—			—		Kepler et al. (2017)
*B.pseudomilitaris*	BCC 2091	Lepidoptera	—			—		Kepler et al. (2017)
*Cordycepsaraneae*	BCC 85066^T^	Arachnid; Araneae						Mongkolsamrit et al. (2020b)
*C.inthanonensis*	BCC 55812^T^	Lepidoptera			—			Mongkolsamrit et al. (2020b)
*C.inthanonensis*	BCC 56302	Lepidoptera						Mongkolsamrit et al. (2020b)
*C.kuiburiensis*	BCC 90322^T^	Araneidae					—	Crous et al. (2019)
*C.militaris*	OSC 93623	Lepidoptera					—	Sung and Spatafora (2004); Spatafora et al. (2007); Kepler et al. (2012)
*C.militaris*	YFCC 6587	Lepidoptera	—					Wang et al. (2020)
*C.nidus*	HUA 186125^T^	Araneidae	—			—		Chirivı´ et al. (2017)
*C.piperis*	CBS 116719	Hemiptera	—					Chaverri et al. (2005); Bischoff et al. (2004); Johnson et al. (2009); Kepler et al. (2017)
*Engyodontiumaranearum*	CBS 309.85	Arachnida	—					Sung et al. (2001); Kepler et al. (2017)
*Flavocilliumbifurcatum*	YFCC 6101^T^	Lepidoptera; Noctuidae	—					Wang et al. (2020)
*Gamszareahumicola*	CGMCC3 19303^T^	Soil				—		Zhang et al. (2020)
*G.humicola*	LC 12462	Soil				—		Zhang et al. (2020)
*Gibellulacebrennini*	BCC 39705	Arachnida; Cebrenninuscf.magnus						Kuephadungphan et al. (2020)
*G.cebrennini*	BCC 53605^T^	Arachnida; Cebrenninuscf.magnus						Kuephadungphan et al. (2020)
G.clavuliferavar.alba	ARSEF 1915	Arachnida				—		Chaverri et al. (2005); Spatafora et al. (2007); Crous et al. (2019)
*G.gamsii*	BCC 25798	Arachnida; Araneida						Kuephadungphan et al. (2019)
*G.gamsii*	BCC 27968^T^	Arachnida; Araneida					—	Kuephadungphan et al. (2019)
*G.scorpioides*	BCC 43298	Arachnida, *Portia* sp.						Kuephadungphan et al. (2020)
*G.scorpioides*	BCC 47976 ^T^	Arachnida, *Portia* sp.						Kuephadungphan et al. (2020)
*Hevansiaarachnophila*	NHJ 2633	Arachnida						Ridkaew et al. Unpublished data (2009); Kuephadungphan Unpublished data (2018)
*H.arachnophila*	NHJ 2465	Arachnida		—				Kuephadungphan Unpublished data (2018); this study
***H.minuta***	BCC 47519^T^	Araneae, *Meotipa* sp.						**This study**
***H.minuta***	BCC 47520	Araneae, *Meotipa* sp.						**This study**
*H.nelumboides*	TNS 16306	Araneidae	—	—		—		Kepler et al. (2017)
*H.novoguineensis*	BCC 2190	Arachnida	—			—	—	Kepler et al. (2017)
***H.novoguineensis***	BCC 42675	Araneae				—		**This study**
***H.novoguineensis***	BCC 49323	Araneae				—		**This study**
*H.novoguineensis*	CBS 610.80^T^	Arachnida				—		Mongkolsamrit et al. (2020b)
H.cf.novoguineensis	BCC 2093	Arachnida	—			—		Kepler et al. (2017)
H.cf.novoguineensis	NHJ 4314	Arachnida	—	—				Johnson et al. (2009)
H.cf.websteri	BCC 23860	Arachnida				—	—	Kuephadungphan et al. (2019)
H.cf.websteri	BCC 36541	Arachnida						Kuephadungphan Unpublished data (2018)
*Hyperdermiumpulvinatum*	P.C. 602	Hemiptera	—				—	Chaverri et al. (2005)
*Jenniferiacinerea*	BCC 2191	Arachnida, *Amyciaea* sp.				—	—	Kuephadungphan et al. (2019)
*J.cinerea*	NHJ 03510^T^	Araneae, *Amyciaea* sp.						Johnson et al. (2009); Ridkaew et al. Unpublished data (2009)
***J.griseocinerea***	BCC 42062^T^	Araneae, *Diaea* sp.						**This study**
***J.griseocinerea***	BCC 42063	Araneae, *Diaea* sp.						**This study**
***J.griseocinerea***	BCC 54893	Araneae, Diaeacf.dorsata				—		**This study**
***J.griseocinerea***	BCC 57821	Araneae, Diaeacf.dorsata				—		**This study**
***J.thomisidarum***	BCC 48932	Araneae, Diaeacf.dorsata				—		**This study**
***J.thomisidarum***	BCC 49257	Araneae, Diaeacf.dorsata				—	—	**This study**
***J.thomisidarum***	BCC 54482	Araneae, Diaeacf.dorsata				—	—	**This study**
***J.thomisidarum***	BCC 66224	Araneae, Diaeacf.dorsata				—		**This study**
***J.thomisidarum***	BCC 37881^T^	Araneae, Diaeacf.dorsata						**This study**
***J.thomisidarum***	BCC 37882	Araneae, Diaeacf.dorsata						**This study**
*Lecanicilliumantillanum*	CBS 350.85^T^	Agaric	—					Sung et al. (2001); Chaverri et al. (2005); Spatafora et al. (2007)
*L.aranearum*	CBS 726.73a	Arachnid, Araneae	—					Sung et al. (2001); Sung et al. (2007)
*Liangiasinensis*	YFCC 3103^T^	*Beauveriayunnanensis*	—					Wang et al. (2020)
*L.sinensis*	YFCC 3104	*Beauveriayunnanensis*	—					Wang et al. (2020)
*Neotorrubiellachinghridicola*	BCC 39684	Orthopterida	—					Thanakitpipattana et al. (2020)
*N.chinghridicola*	BCC 80733^T^	Orthopterida	—		—			Thanakitpipattana et al. (2020)
*Parahevansiakoratensis*	NHJ 666.01	Arachnida				—	—	Ridkaew et al. Unpublished data (2009)
*Pa.koratensis*	NHJ 2662	Lepidoptera						Ridkaew et al. Unpublished data (2009); this study
*Pleurodesmosporalepidopterorum*	DY 10501^T^	Lepidoptera		—				Chen et al. (2021b)
*P.lepidopterorum*	DY 10502	Lepidoptera		—		—		Chen et al. (2021b)
***Polystromomycesaraneae***	BCC 93301^T^	Arachnida						This study
*Pseudogibellulaformicarum*	BCC 84257	*Ophiocordycepsflavida*					—	Mongkolsamrit et al. (2021)
*P.formicarum*	CBS 433.73	*Pahothyreustarsatus*					—	Vu et al. (2019); Mongkolsamrit et al. (2021);
*Samsoniellaaurantia*	TBRC 7271^T^	Lepidoptera						Mongkolsamrit et al. (2018)
*S.aurantia*	TBRC 7272	Lepidoptera				—		Mongkolsamrit et al. (2018)
*Simplicilliumlanosoniveum*	CBS 704.86	*Hemileiavastatrix*	—					Sung et al. (2001); Spatafora et al. (2007)
*S.lanosoniveum*	CBS 101267	*Hemileiavastatrix*	—					Sung et al. (2001); Spatafora et al. (2007)

The accession numbers marked in bold font refer to sequences new in this study or have been generated by our group in Thailand. ^T^ex-type species.

### ﻿Sequence alignment and phylogenetic analyses

The DNA sequences generated in this study were examined for ambiguous bases and corrected using BioEdit v. 7.2.5 (Hall 1999), then submitted to GenBank. Sequences of ITS, LSU, *tef1*, *rpb1* and *rpb2*, of closely-related taxa for the analyses were taken from previous studies as shown in Table 1. The phylogenetic analyses for combined and single-locus alignments were performed using RAxML-HPC2 on XSEDE v. 8.2.12 (Stamatakis 2014) in CIPRES Science Gateway portal, with GTRGAMMA+I model and 1000 bootstrap iterations. Bayesian Inference (BI) of the phylogenetic relationship was performed in MrBayes v. 3.2.7a (Ronquist et al. 2012), with best-fit models selected using MrModeltest v. 2.2 (Nylander 2004). The best model was GTR + G + I. Markov Chain Monte Carlo (MCMC) simulations were run for 2,000,000 generations, sampling every 1000 and discarding the first 10% as burn-in. The remaining 20,001 trees were used to calculate the posterior probability values. RAxML and BI output were imported into TreeView v. 1.6.6 to visualise the phylogenetic tree (Page 1996).

## ﻿Results

### ﻿Molecular phylogeny

**Figure 1.**
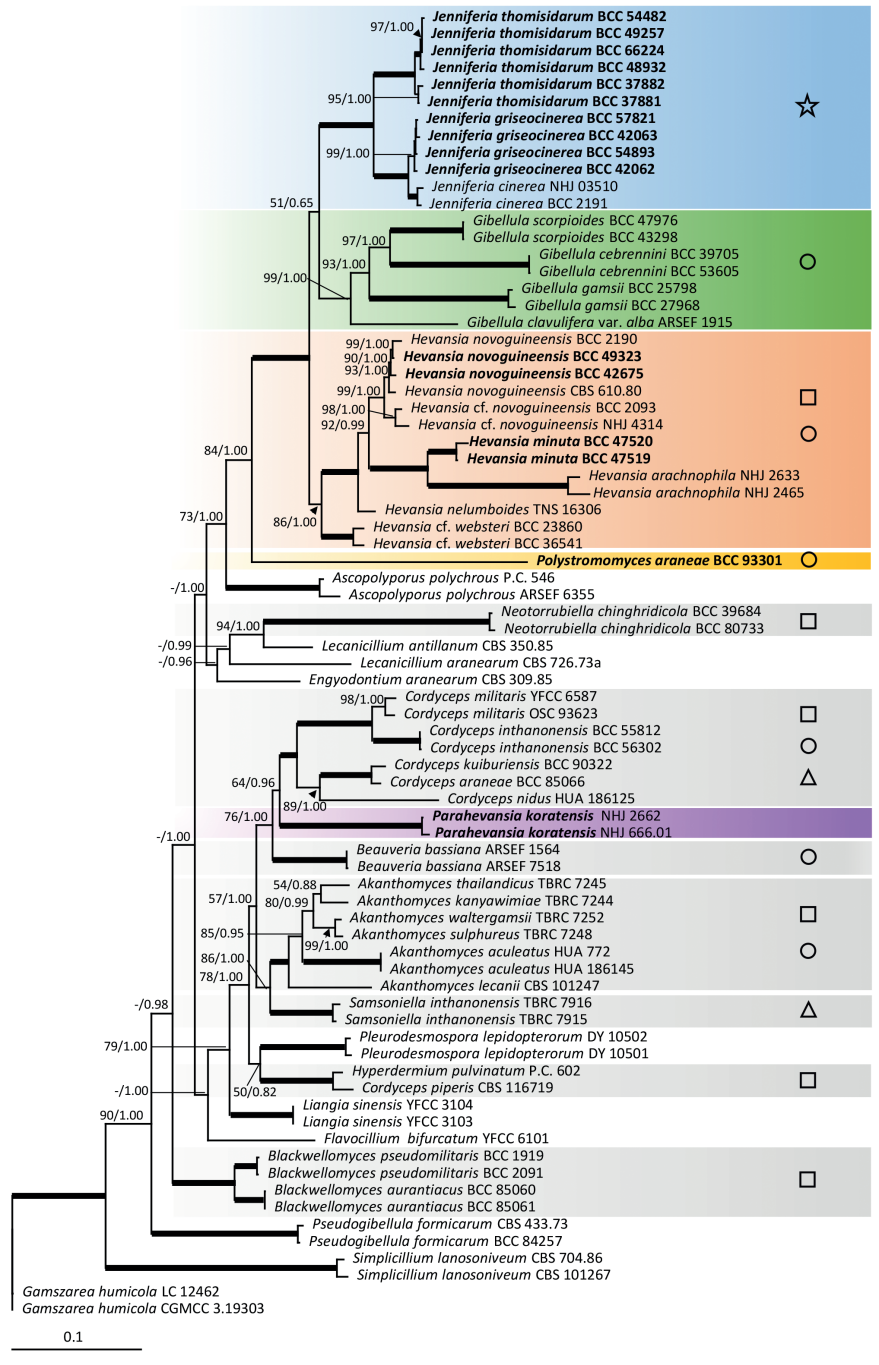
RAxML tree of *Hevansia*, *Jenniferia*, *Parahevasia*, *Polystromomyces* and related genera in the Cordycipitaceae from a combined ITS, LSU, *tef1*, *rpb1* and *rpb2* dataset. Numbers at the major nodes represent Maximum Likelihood Bootstrap (MLB) and Bayesian Posterior Probabilities (BPP). Bold lines in the tree represent 100% of MLB and 1.00 of BPP. Symbols on the right-hand side correspond to the types of ascospore morphologies found in each genus that are observed in natural specimens of Cordycipitaceae described in Fig. 2.

We generated 65 new sequences (15 ITS, 15 LSU, 15 *tef1*, 7 *rpb1* and 13 *rpb2*) from living cultures (Table 1). *Gamszareahumicola* Z.F. Zhang & L. Cai (3.19303 and LC 12462) was used as an outgroup. The combined dataset from 77 specimens, with multi-locus sequences totalling an alignment length of 4231 characters with gaps (ITS 656, LSU 841, *tef1* 921, *rpb1* 764 and *rpb2* 1049) was analysed. The maximum-likelihood phylogenetic analyses resulted in a multi-locus tree with maximum likelihood bootstrap values (MLB) shown in Fig. 1 and in single-locus trees (Suppl. material 1: Figs S1–S5). The nodes were also evaluated with Bayesian posterior probabilities (BPP). Bold lines in the tree represent 100% of MLB and 1.00 of BPP.

The phylogenetic analyses supported *Hevansia* as a monophyletic clade with maximum support (MLB = 86 / BPP = 1.00), including ex-type *H.novoguineensis* (CBS 610.80) from Papua New Guinea as the type species. The strain BCC 42675, isolated from a sexual morph from Thailand, clustered with *H.novoguineensis* (CBS 610.80) with high support (MLB = 93 / BPP = 1.00), revealing a sexual morph connection to this species. Two strains from Thailand (BCC 2093, NHJ 4314) formed a sister clade to the clade containing the ex-type strain of *H.novoguineensis* with maximum support for the separating node (MLB = 98 / BPP = 1.00). This separation was observed with the phylogenetic signal from only LSU, *tef1*, while the other markers either did not have sufficient sample coverage for comparison (ITS, *rpb1*: Suppl. material 1: Figs S1 and S4) or did not recover this separation (*rpb2*: Suppl. material 1: Fig. S5). These two specimens were thus named as H.cf.novoguineensis herein. Two unknown *Hevansia* strains from both an asexual state (BCC 47520) and a sexual state (BCC 47519) were found as a well-supported clade (MLB = 100 / BPP = 1.00) within *Hevansia*, but separated from *H.novoguineensis*, which was also recovered by all single-locus phylogenies. These two *Hevansia* strains were thus proposed as a new species, *Hevansiaminuta*. Furthermore, two strains of *H.arachnophila* (NHJ 2465, NHJ 2633) and two strains of H.cf.websteri (BCC 23860, BCC 36541) were included in our phylogenetic analyses and shown to belong to *Hevansia*. Additionally, a strain formerly named as *Hevansiakoratensis* (NHJ 666.01 (BCC 1485)) and a strain previously recognised as *H.websteri* (NHJ 2662 (BCC 2113)) formed together an independent clade with strong support (MLB = 100/ BPP = 1.00), out of the *Hevansia* clade and in the proximity of *Cordyceps* species. Hence, this clade does not belong to *Hevansia* and is proposed as a new genus named *Parahevansia* (Fig. 1).

The combined-genes phylogenetic tree revealed one important terminal monophyletic clade close to *Gibellula* with total support (MLB = 100 / BPP = 1.00), Fig. 1. This clade is proposed as a new genus named *Jenniferia*. The genus *Jenniferia* formed a monophyletic clade separated from *Hevansia* and *Gibellula* for all the markers used in this study (Suppl. material 1: Figs S1–S5). *Jenniferia* contains two novel species, *Jenniferiagriseocinerea* and *J.thomisidarum* and includes *J.cinerea*, which is proposed as a new combination of *H.cinerea* to this genus. *Jenniferiagriseocinerea* is distinguished from *J.cinerea*, based on the separated monophyletic clades in the multi-locus phylogeny (Fig. 1). The separation between the two species was recovered in most of the single-locus phylogenies (*tef1*, *rpb1* and *rpb2*, but not ITS nor LSU: Suppl. material 1: Figs S1–S5).

The combined-genes analysis also revealed a deep taxon from a unique specimen (BCC 93301), branched as sister to the three genera occurring on spider egg sac (*Gibellula*, *Hevansia* and *Jenniferia*), which was thus proposed as a new genus *Polystromomyces*. The branching of this specimen had high support (MLB = 84 / BPP = 1.00) and was found consistently amongst different markers (Suppl. material 1: Figs S1–S5). This taxon was never within the three main genera occurring on spiders (*Gibellula*, *Hevansia* and *Jenniferia*), supporting the status of a different genus. *Polystromomyces* contains a new species, *Po.araneae*.

### ﻿Overview of types of ascospores in Cordycipitaceae

**Figure 2.**
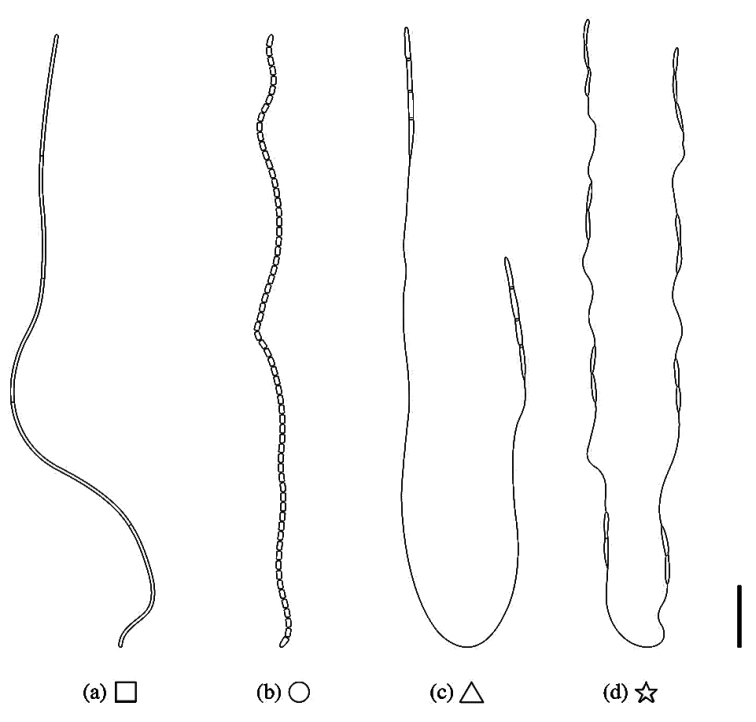
The types of ascospores morphologies observed in natural specimens of Cordycipitaceae: **a** filiform, multiseptate, whole ascospores (square) **b** filamentous, multiseptate ascospores disarticulating into part-spores (circle) **c** bola-shaped, whole ascospores (triangle) and **d** whole ascospores with septate part-spores alternately connected with thread-like structures (star). Scale bars: 10 µm (**a, b**); 20 µm (**c, d**).

Different types of ascospore morphologies were observed in natural specimens of Cordycipitaceae as shown in Fig. 2. Three types observed previously include: (a) filiform, multiseptate, whole ascospores, (b) filamentous, multiseptate ascospores disarticulating into part-spores and (c) bola-shaped, whole ascospores, non-disarticulating, characterised by a thread-like structure connected to fusiform, terminal, multi-septate parts at both ends, resembling a skipping rope. We observed a new type of ascospore morphology in *Jenniferia* as shown in Fig. 2(d), in which septate part-spores are alternately connected with thread-like structures along the whole ascospore. The ascospore morphologies shown in Fig. 2a, b and d were observed on spider-pathogenic fungi in this study.

### ﻿Taxonomy

#### ﻿ Hevansia

Taxon classificationFungiHypocrealesCordycipitaceae

Luangsa-ard, Hywel-Jones & Spatafora, in Kepler, Luangsa-ard, Hywel-Jones, Quandt, Sung, Rehner, Aime, Henkel, Sanjuan, Zare, Chen, Li, Rossman, Spatafora, Shrestha, IMA Fungus 8: 348 (2017). Emend. S. Mongkolsamrit, W. Noisripoom & K. Tasanathai

225C50F2-9050-5AEE-87B9-9F07392DE3AC

≡ Akanthomycesnovoguineensis Samson & B.L. Brady, Trans. Br. mycol. Soc. 79: 571 (1982).

##### Type species.

*Hevansianovoguineensis* (Samson & B.L. Brady) Luangsa-ard, Hywel-Jones & Spatafora, IMA Fungus 8: 349 (2017).

##### Emended generic description

**(modified from Kepler et al. 2017).***Circumscription*: The sexual morph characteristics in genus are emended, based on three species *H.minuta*, *H.nelumboides* and *H.novoguineensis* producing sexual morph as members of *Hevansia* lineage in Fig. 1. Sexual morph: *Stromata* arising from dorsal abdomen, stipe 1–10 mm, fertile part at the terminal of stipe, ca. 1–3 × 1–2 mm, white to cream. *Perithecia* immersed, narrowly ovoid. *Asci* cylindrical with thickened caps, 8-spored, ascospores hyaline, filiform, whole or disarticulating into part-spores. Asexual morph: *Synnemata* erect, simple or branched, solitary to numerous, cylindrical to clavate, mycelium covering host, white, cream to ash-grey or brownish-white. *Phialides* in a monolayer, sparsely scattered or crowded, on mycelium or on a basal cell, smooth-walled, cylindrical, globose, obovoid, with distinct necks. *Conidia* one-celled, smooth-walled, hyaline, occasionally in a short chain, clavate, cylindrical, fusiform to narrowly obclavate. Colony on PDA white, reverse cream, orange to pale red. Some species produce pale red pigment diffusing in the medium.

##### Notes.

Two specimens of *H.arachnophila* (NHJ 2465, NHJ 2633) were described by Hywel-Jones (1996). While the type strain of *H.websteri* (NHJ 2661) and living cultures are unavailable, available sequences of *H.arachnophila* and two strains of *H.websteri* (BCC 23860, BCC 36541) were retrieved from the GenBank nucleotide database and used in this study. The phylogenetic tree revealed that *H.arachnophila* and *H.websteri* (BCC 23860, BCC 36541) belong to the genus *Hevansia* (Fig. 1). The two strains of *H.websteri* (BCC 23860, BCC 36541) were not designated as type, nor as neotype. These strains (BCC 23860, BCC 36541) were thus named as Hevansiacf.websteri. *Hevansialongispora* and *H.ovalongata* were not included in the phylogenetic study because multi-locus sequence data are unavailable. To better resolve the genus *Hevansia*, *H.longispora*, *H.ovalongata* and *H.websteri* should be recollected from the locality and designated as neotypes and studied for their phylogenetic affinity to other *Hevansia* species in the future. However, *H.longispora*, *H.ovalongata* and *H.websteri* were accepted in *Hevansia* following complete and well-illustrated descriptions by Hywel-Jones (1996), Hsieh et al. (1997) and Huang et al. (2000).

#### ﻿ Hevansia novoguineensis

Taxon classificationFungiHypocrealesCordycipitaceae

(Samson & B.L. Brady) Luangsa-ard, Hywel-Jones & Spatafora

A576212B-CED9-5739-8231-AFC78C231622

Fig. 3

##### Remark.

The description below is based on natural specimens collected in Thailand.

##### Description.

Spider hosts covered by light yellow to pale yellow (158A–B) mycelium. Sexual morph: *Stromata* stipitate, solitary or multiple. *Stipes* cylindrical, arising from the dorsal region of the host, white to pale yellow, 3–5 mm long, 0.5–1 mm broad. *Fertile heads* produce at the terminal of stipes, disc-shaped, upper surface slightly convex, 1–3 × 1–2 mm. *Perithecia* completely immersed, narrowly ovoid, 500–750 × 200–300 µm, ostioles strong orange yellow (163B). *Asci* cylindrical, 8-spored, 350–450 µm long, 5–7 µm broad, with cap 3–5 µm thick. *Ascospores* hyaline, filiform, whole ascospores, 400–460 × 1–1.5 µm. Asexual morph: *Synnemata* multiple, cylindrical, occasionally acuminate apex, white, up to 8 mm long, 50–200 µm broad. *Conidiogenous cells* phialidic, scattered along with the synnemata. *Phialides* solitary, globose to subglobose, arising from the mycelium, (4)5–5.5(6) × (4)5–5.5(6) µm, with distinct necks, 0.5–1.5 × 0.5–1 µm. *Conidia* hyaline, fusoid or fusiform-elliptical, (2)6–8(10) × 1–2(2.5) µm.

**Figure 3.**
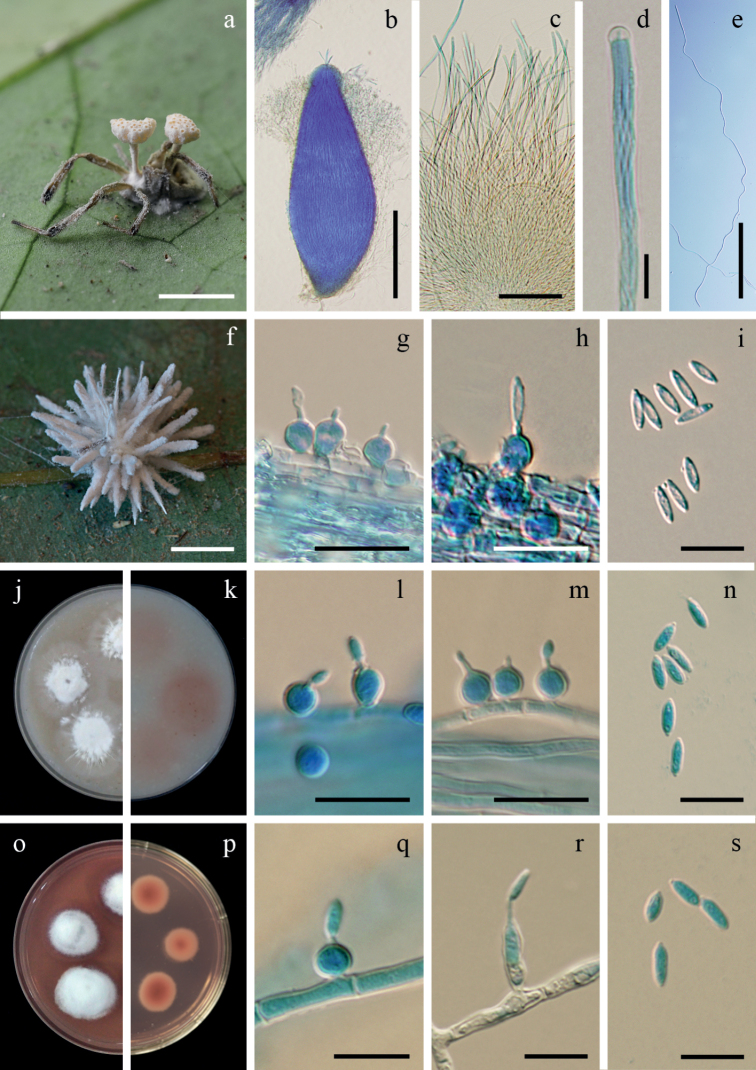
*Hevansianovoguineensis***a** fungus on a spider (BBH 32171) **b** perithecium **c** asci **d** ascus tip **e** filiform, whole ascospore **f** fungus on a spider (BBH 31299) **g–i** phialides with conidia on synnema **j, k** colonies on OA at 21 days (**j** obverse, **k** reverse) **l–n** phialides with conidia on OA**o, p** colonies on PDA at 21 days with purplish-red pigment diffusing in agar medium (**o** obverse, **p** reverse) **q–s** phialides with conidia on PDA. Scale bars: 5 mm (**a, f**); 200 µm (**b**); 100 µm (**c, e**); 10 µm (**d, g–i, l–n, q, r, s**).

##### Culture characteristics.

Colonies on OA attaining a diam. of 18–20 mm in 21 days, cottony with high mycelium density in the middle of colonies, mycelium with low density around the margin of colonies, flattened, white, reverse deep pink (180D). Sparse synnemata with conidiogenous cells producing conidia observed after 30 days, white, on the edge of a colonies. *Phialides* solitary, globose to subglobose, (4)5.5–6.5(7) × 3.5–5(5.5) µm, distinct necks, 1–3 × 0.5–1 µm. *Conidia* hyaline, fusoid, fusiform-elliptical, (2)6–10(13) × 1–3 µm.

Colonies on PDA attaining a diam. of 7–9(10) mm in 21 days, cottony with high mycelium density, white, moderate purplish-red to dark purplish-pink (186B–C) pigment diffusing in the medium, reverse moderate red (180 A–B). Sporulation observed after 30 days with absence of synnemata. *Phialides* arising from aerial hyphae, solitary, mostly globose to subglobose, occasionally cylindrical, (4)5.5–11.5(15) × 2–3.5(5) µm, distinct necks, 0.5–2 × 0.5–1 µm. *Conidia* hyaline, fusoid, fusiform-elliptical, cylindrical, (2)6–9.5(11) × 1–3 µm.

##### Host.

Spiders (Araneae, Theridiidae).

##### Habitat.

Specimens were found on the underside of dicot leaves of forest plants.

##### Materials examined.

Thailand, Nakhon Ratchasima Province, Khao Yai National Park, 14°26'20.72"N, 101°22'20.02"E, on spider (Web builder, Araneae) attached to the underside of a dicot leaf of forest plants, 10 June 2010, K. Tasanathai, P. Srikitikulchai, S. Mongkolsamrit, R. Ridkaew, MY6026.01 (BBH 32171, BCC 42675) isolated from ascospores; idem, 6 April 2010, K. Tasanathai, S. Mongkolsamrit, T. Chohmee, A. Khonsanit, R. Ridkaew, MY6988.01 (BBH 31299, BCC 49323) isolated from conidia; Kamphaeng Phet, Khlong Lan National Park, 16°7'46.84"N, 99°16'53.11"E, on spider (Web builder, Araneae, Theridiidae) attached to the underside of a dicot leaf of forest plants, 6 November 2007, K. Tasanathai, S. Mongkolsamrit, P. Srikitikulchai, B. Thongnuch, R. Ridkaew, A. Khonsanit, W. Chaygate, MY2770 (BBH 22744, BCC 28581), MY2771 (BBH 22745, BCC 28582), MY2775 (BBH 22747, BBC 28585).

##### Notes.

*Hevansianovoguineensis* is morphologically similar to *H.nelumboides*, both species producing fertile heads at the terminal end of stipes. The perithecia are completely immersed. However, *H.novoguineensis* differs from *H.nelumboides* in producing whole ascospores. *Hevansianelumboides* produces multiseptated ascospores disarticulating into part-spores (Kobayasi and Shimizu 1977; Shimizu 1994). Based on natural specimens, the conidia from Thai specimens are shorter than those reported for specimens from Papua New Guinea (2–10 × 1–2.5 µm vs. 10.5–17.5 × 1.5–3 µm) (Samson and Brady 1982). In addition, there are other species producing the fertile heads at the terminal end of stipes infecting ants (Hymenoptera), for example, *Ophiocordycepsbinata* (H.C. Evans & Samson) J.P.M. Araújo, H.C. Evans & D.P. Hughes, *O.pseudolloydii* (H.C. Evans & Samson) G.H. Sung, J.M. Sung, Hywel-Jones & Spatafora and *O.lloydii* (H.S. Fawc.) G.H. Sung, J.M. Sung, Hywel-Jones & Spatafora (Araújo et al. 2020). *Ophiocordycepsbinata* is most similar to *H.novoguineensis* by producing disc-shaped fertile heads, while fertile heads in *O.pseudolloydii* and *O.lloydii* are subglobose.

### ﻿Currently accepted species of *Hevansia*

#### ﻿ Hevansia arachnophila

Taxon classificationFungiHypocrealesCordycipitaceae

(Petch) Luangsa-ard, Hywel-Jones & Spatafora, IMA Fungus 8: 348 (2017).

EF3845AA-278A-5760-A8DF-E72428F2B4B4

≡ Trichosterigmaarachnophilum Petch [as ‘arachnophila’], Trans. Br. mycol. Soc. 8: 215 (1923).
≡ Hirsutellaarachnophila (Petch) Petch, Trans. Br. mycol. Soc. 9: 93 (1923).
≡ Akanthomycesarachnophilus (Petch) Samson & H.C. Evans, Acta bot. neerl. 23: 33 (1974).

#### ﻿ Hevansia longispora

Taxon classificationFungiHypocrealesCordycipitaceae

(B. Huang, S.B. Wang, M.Z. Fan & Z.Z. Li) Luangsa-ard, Hywel-Jones & Spatafora, IMA Fungus 8: 349 (2017).

97F91D8A-BB49-5780-830B-C98A6F04CB77

≡ Akanthomyceslongisporus B. Huang, S.B. Wang, M.Z. Fan & Z.Z. Li, Mycosystema 19: 172 (2000).

#### ﻿ Hevansia nelumboides

Taxon classificationFungiHypocrealesCordycipitaceae

(Kobayasi & Shimizu) Luangsa-ard, Hywel-Jones & Spatafora, IMA Fungus 8: 349 (2017).

AD4FB252-0B1D-57F4-A55B-2262A48798D2

≡ Cordycepsnelumboides Kobayasi & Shimizu, Kew Bull. 31: 557 (1977).

#### ﻿ Hevansia ovalongata

Taxon classificationFungiHypocrealesCordycipitaceae

(L.S. Hsieh, Tzean & W.J. Wu) Luangsa-ard, Hywel-Jones & Spatafora, IMA Fungus 8: 349 (2017).

C42167C3-2222-5179-B209-B09C3EF0000B

≡ Akanthomycesovalongatus L.S. Hsieh, Tzean & W.J. Wu, Mycologia 89: 321 (1997).

#### ﻿ Hevansia websteri

Taxon classificationFungiHypocrealesCordycipitaceae

(Hywel-Jones) Luangsa-ard, Hywel-Jones & Spatafora, IMA Fungus 8: 349 (2017).

6CBB45B2-7A32-583D-AF41-C8EB28BAC6EA

≡ Akanthomyceswebsteri Hywel-Jones, Mycol. Res. 100: 1068 (1996).

#### ﻿ Hevansia minuta

Taxon classificationFungiHypocrealesCordycipitaceae

Tasanathai, Noisripoom & Mongkolsamrit sp. nov.

ECA6F486-376A-5132-898F-534D03CD1CFE

 843088

Fig. 4

##### Typification.

Thailand, Chumphon Province, Heo Lom Waterfall, 9°43'45.04"N, 98°40'52.71"E, on spider (Web builder, Araneae, Theridiidae, *Meotipa* sp.) attached to the underside of a dicot leaf of forest plants, 30 May 2011, K. Tasanathai, P. Srikitikulchai, A. Khonsanit, K. Sansatchanon, D. Thanakitpipattana, MY6537.01 (BBH 30490, holotype), ex-type culture BCC 47519 isolated from ascospores.

**Figure 4.**
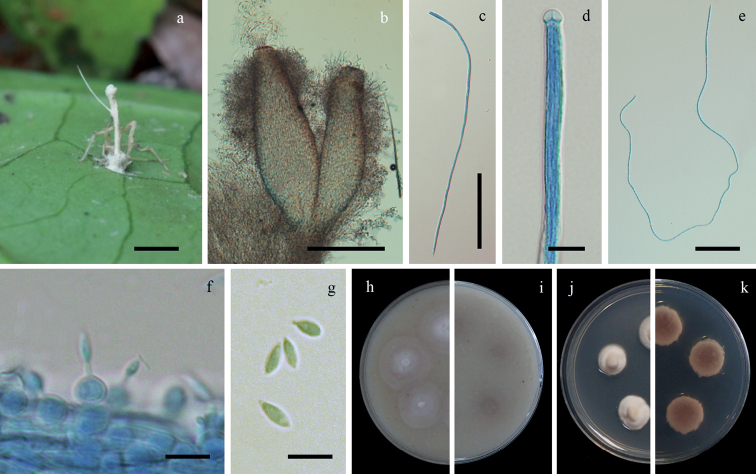
*Hevansiaminuta***a** fungus on a spider (BBH 30490) **b** perithecia **c** ascus **d** ascus tip **e** filiform, whole ascospore **f, g** phialides with conidia **h, i** colonies on OA at 21 days (**h** obverse, **i** reverse) **j, k** colonies on PDA at 21 days (**j** obverse, **k** reverse). Scale bars: 5 mm (**a**), 100 µm (**b**), 50 µm (**c**), 10 µm (**d, f, g**), 20 µm (**e**).

##### Etymology.

Refers to the small stroma of this species.

##### Description.

Spider host covered by white mycelium. Sexual morph: *Stromata* stipitate, arising from the dorsal region of the host, solitary, cylindrical to enlarging apically, white to cream, 10 mm long, 1 mm broad. *Fertile head* oval, ca. 2–2.5 mm long, ca. 1.5 mm broad. *Perithecia* completely immersed, narrowly ovoid, 400–500 × 100–170 µm. *Asci* cylindrical, 8-spored, 325–450 × 3–5 µm, with cap 2–5 µm thick. *Ascospores* hyaline, filiform, whole ascospores, 320–450 × 0.5–1.5 µm. Asexual morph: *Conidiogenous cells* phialidic scattered along with the stipe. *Phialides* solitary, globose to ovoid, arising from the mycelium, 5–7 × 5–6 µm, distinct necks, 1–2 × 0.5–1 µm. *Conidia* hyaline, fusiform, 2–7 × 2–3 µm.

##### Culture characteristics.

Colonies on OA attaining a diam. of 15–18(20) mm in 21 days, cottony with high mycelium density, white. *Conidia* and reproductive structures not observed.

Colonies on PDA attaining a diam. of 8–9(10) mm in 21 days, cottony with high mycelium density, white, reverse pale yellow (161C–D). *Conidia* and reproductive structures not observed.

##### Host.

Spiders (Araneae, Theridiidae, *Meotipa* sp.).

##### Habitat.

Specimens were found on the underside of dicot leaves of forest plants.

##### Additional materials examined.

Thailand, Chumphon Province, Heo Lom Waterfall, 9°43'45.04"N, 98°40'52.71"E, on spider (Web builder, Araneae, Theridiidae, *Meotipa* sp.) attached to the underside of a dicot leaf of forest plants, 30 May 2011, K. Tasanathai, P. Srikitikulchai, A. Khonsanit, K. Sansatchanon, D. Thanakitpipattana, MY06537.02 (BBH 30490, paratype), ex-paratype culture BCC 47520 isolated from conidia.

##### Notes.

*Hevansiaminuta* differs significantly from *H.novoguineensis* and *H.nelumboides* in the shape of the fertile heads, which is oval in *H.minuta* and disc-shaped, slightly convex on the upper surface in *H.novoguineensis* and *H.nelumboides*. Additionally, *H.minuta* differs from *H.novoguineensis* in the size of the perithecia. In *H.minuta*, perithecia are smaller than those reported for *H.novoguineensis* (400–500 × 100–170 µm vs. 500–750 × 200–300 µm) (Table 2). Synnema in *H.minuta* was not observed in the natural specimen, while the other species in *Hevansia* produce synnemata (Table 3). *Hevansiaminuta* does not produce pigment in culture. Meanwhile, *H.novoguineensis* produces a purplish-red pigment diffusing in PDA plates.

**Table 2.** Morphological comparisons of sexual morphs in *Hevansia*, *Jenniferia* and *Polystromomyces*.

Species	Host	Stromata	Fertile part	Perithecia	Asci	Ascospores	References
*Hevansiaminuta*	Spider (Theridiidae, *Meotipa* sp.)	Stipitate, solitary, white to cream, 10 mm long, 1 mm broad	Oval, ca. 2–2.5 mm long, ca. 1.5 mm broad	Immersed, narrowly ovoid, 400–500 × 100–170 µm	Cylindrical, 325–450 × 3–5 µm	Filiform, whole ascospores, 320–450 × 0.5–1.5 µm	This study
*H.nelumboides*	Spider	Stipitate, white, 4 mm long, 0.4 mm broad	Disc-shaped, 2 × 0.8 mm	Immersed, fusoid-ellipsoidal, 535–545 × 180–190 µm	400–450 × 5–6 µm	Part-spores, ca. 5 × 1 µm	Kobayasi and Shimizu (1977)
*H.novoguineensis*	Spider (Theridiidae)	Stipitate, solitary, or multiple, cylindrical, white to pale yellow, 3–5 mm long, 0.5–1 mm broad	Disc-shaped, upper surface slightly convex, 1–3 × 1–2 mm	Immersed, narrowly ovoid, 500–750 × 200–300 µm	Cylindrical, 350–450 × 5–7 µm	Filiform, whole ascospores, 400–460 × 1–1.5 µm	This study
*Jenniferiagriseocinerea*	Spider (Thomisidae, Diaeacf.dorsata, *Diaea* sp.)	Non-stipitate	Perithecia aggregated in clusters forming a cushion	Superficial, ovoid, 650–850 × 250–320 µm	Cylindrical, 375–460 × 5–6 µm	Whole ascospores with septate part-spores alternately connected with thread-like structures, up to 400 µm long, each cell narrowly fusiform, 10–15 × 1–2 µm, filiform regions, 35–45 × 0.2–0.8 µm	This study
*J.thomisidarum*	Spider (Thomisidae, Diaeacf.dorsata)	Non-stipitate	Perithecia aggregated in clusters forming a cushion	Superficial, obpyriform, 850–1100 × 300–400 µm	Cylindrical, 520–700 × 4–6 µm	Whole ascospores with septate part-spores alternately connected with thread-like structures, up to 680 µm long, each cell narrowly fusiform, 10–20 × 1–2 µm, filiform regions, 30–50 × 0.2–0.8 µm	This study
*Polystromomycesaraneae*	Spider egg sac	Stipitate, multiple, moderate yellow, 8–12 mm long, 1–3 mm broad	Disc-shaped, upper surface slightly convex, 3–4 × 2–3.5 mm	Immersed, narrowly ovoid, 1000–1400 × 200–350 µm	Cylindrical, 400–1000 µm long, 3.5–6 µm	Part-spores, cylindrical, 2–6 × 1–3 µm	This study

**Table 3.** Morphological comparisons of asexual morphs in *Hevansia*, *Jenniferia* and *Parahevansia*.

Species	Host	Synnemata	Phialides	Conidia	References
*Hevansiaarachnophila*	Spider	Simple, solitary (rarely two or three together), cylindrical, cream, up to 6 mm long, 45–100 µm broad	Globose, 3–4.5 µm broad, with distinct necks, 1–2 × 0.5 µm	Cymbiform, 3.5–6 × 1–1.5 µm	Hywel-Jones (1996)
*H.longispora*	Spider	Multiple, clavate, brown, 250–700 µm long	Ellipsoid to cylindrical, 7–15 × 2–4 µm	Cylindrical to fusiform, 8.8–14.8 × 2–3 µm	Huang et al. (2000)
*H.minuta*	Spider (Theridiidae, *Meotipa* sp.)	Non-synnemata	Globose to ovoid, 5–7 × 5–6 µm with distinct necks, 1–2 × 0.5 µm	Fusiform, 2–7 × 2–3 µm	This study
*H.nelumboides*	Spider	NA	Elongate	Ovoid, 5 × 3 µm	Kobayasi and Shimizu (1977)
*H.novoguineensis*	Spider (Theridiidae)	Multiple, cylindrical, occasionally acuminate apex, white, up to 8 mm long, 50–200 µm broad	Globose to subglobose, 4–6 × 4–6 µm, with distinct necks, 0.5–1.5 × 0.5–1 µm	Fusoid or fusiform-elliptical, 2–10 × 1–2.5 µm	This study
*H.novoguineensis*	Spider	Multiple, slender, acuminate apex, white to pale yellow, 3.5 mm long, 50–150 µm broad	Globose to ovoid, 5–6.5 × 4–6 µm broad, with distinct necks, 2–3 × 0.8–1.5 µm	Cylindrical, curved or slightly fusiform, 10.5–17.5 × 1.5–3 µm	Samson and Brady (1982)
*H.ovalongata*	Spider	Multiple, simple, or branch, white to greyish-orange, 2.2–9 mm long, 112–520 µm broad	Globose to subglobose, cylindrical, or ellipsoid, 6–8.7 × 4–6.4 µm, with distinct necks, 1.4–3.2 × 0.8–1.8 µm	Ellipsoid, obovate to oblong, 6–10.3 × 2.4–4.4 µm	Hsieh et al. (1997)
*H.websteri*	Spider	Simple, cylindrical, cream-white, up to 12 mm long, 50–70 µm broad	Ellipsoid, 4.5–8.5 × 2–3.5 µm, with distinct necks, 1.5–3 × 0.5 µm	Cylindrical, 4–7 × 1–1.5 µm	Hywel-Jones (1996)
*Jenniferiacinerea*	Spider (Thomisidae, *Amyciaea* sp.)	Multiple, clavate, grey, up to 3 mm long, 60–70 µm broad	Cylindrical, 3.5–6.5 × 1.5–2 µm, with distinct necks, 2–2.5 × 0.5 µm	Clavate, 3.5–5.5 × 1–1.5 µm	Hywel-Jones (1996)
*J.griseocinerea*	Spider (Thomisidae, Diaeacf.dorsata, *Diaea* sp.)	Two types of synnemata, long synnemata, cylindrical with blunt end, grey to pale brown, 2.5–5 mm long, 100–150 µm broad, middle of long synnemata, 50–80 μm broad; short synnemata, cylindrical, pale grey to dark grey, up to 450 µm long, 20–50 µm broad	Flask-shaped, 5–10 × 3–5 µm, with distinct necks, 2–3.5 × 0.5–1 µm	Fusiform, 3–6 × 1–2 µm	This study
*J.thomisidarum*	Spider (Thomisidae, Diaeacf.dorsata)	Multiple, cylindrical to clavate, greyish-brown, up to 800 µm long, 30–100 µm broad	Cylindrical, 7–16 × 2–5 µm, with distinct necks, 1–5 × 1–1.5 µm	Fusiform, cylindrical, 3–12 × 1–3 µm	This study
*Parahevansiakoratensis*	Spider (Salticidae)	Multiple, simple, brown at the sterile base, becoming grey white, up to 6 mm long, 50 µm broad	Obovoid to ellipsoid, 4–5.5 × 3–3.5 µm, with distinct necks, 2.5–3 × 0.5–1 µm	Clavate, 4.5–5.5 × 1–1.5 µm	Hywel-Jones (1996)

NA, information not provided in the original description.

### ﻿Key to the species of *Hevansia*

Based on sexual state characters

**Table:** 

1	Ascospores filamentous, disarticulating into part-spores, immersed perithecia, solitary or multiple stipes	***H.nelumboides***
–	Ascospores filiform, whole ascospores, immersed perithecia, solitary or multiple stipes	**2**
2	Ascospores 320–450 × 0.5–1.5 µm, solitary stipe	***H.minuta***
–	Ascospores 400–460 × 1–1.5 µm, solitary or multiple stipes	***H.novoguineensis***

Based on asexual state characters

**Table:** 

1	Phialides mostly arising from the mycelium, globose to subglobose	**2**
–	Phialide arising on basal cells, obovoid, ellipsoid, cylindrical	**3**
2	Conidia cymbiform, 3.5–6 × 1–1.5 µm	***H.arachnophila***
–	Conidia fusiform, 2–7 × 2–3 µm	***H.minuta***
–	Conidia cylindrical, fusoid, fusiform-elliptical, (from Thailand, 2–10 × 1–2.5 µm); occasionally curved, (Papua New Guinea, 10.5–17.5 × 1.5–3 µm	***H.novoguineensis***
–	Conidia oblong, obovate or broadly ellipsoidal 6–10.3 × 2.4–4.4 µm	***H.ovalongata***
3	Conidia cylindrical to fusiform 8.8–14.8 × 2–3 µm	***H.longispora***
–	Conidia cylindrical, 4–7 × 1–1.5 µm	***H.websteri***

#### ﻿ Jenniferia

Taxon classificationFungiHypocrealesCordycipitaceae

Mongkolsamrit, Noisripoom & Tasanathai gen. nov.

2227B7ED-AEED-5B7E-9122-8FD0A7A83CC7

 843089

##### Type species.

*Jenniferiathomisidarum* Mongkolsamrit, Noisripoom & Tasanathai.

##### Etymology.

In honour of Dr. Jennifer Luangsa-ard, for her support and guidance in arthropod pathogenic fungi research.

##### Description.

Spider hosts covered with pale yellow to dark greyish-yellow mycelium. Sexual morph: *Stromata* non-stipitate. *Perithecia* growing in subiculum, superficial, aggregated in clusters forming a cushion. *Asci* cylindrical with thickened caps. *Ascospores* hyaline, septate part-spores alternately connected with thread-like structures along the whole ascospore (Fig. 2d). Asexual morph: *Synnemata* arising from all parts of host, numerous, cylindrical to clavate. *Conidiogenous cells* phialidic, producing along the synnemata or upper part of the synnemata. *Phialides* flask-shaped with distinct necks. *Conidia* hyaline, fusiform or cylindrical.

##### Notes.

*Jenniferia* is strongly supported as a monophyletic clade by having unique morphological characteristics of perithecia and ascospores. In sexual morph specimens, this genus produces aggregated superficial perithecia forming a cushion with septate part-spores alternately connected with thread-like structures along the whole ascospore (Fig. 2d), which are not seen in any allied genera of the family.

#### ﻿ Jenniferia cinerea

Taxon classificationFungiHypocrealesCordycipitaceae

(Hywel-Jones) Mongkolsamrit & Noisripoom comb. nov.

8CFCE167-3F92-5435-A4AF-DA39A3B346E5

 843090

Fig. 5

≡ Akanthomycescinereus Hywel-Jones, Mycol. Res. 100: 1068 (1996).
≡ Hevansiacinerea (Hywel-Jones) Luangsa-ard, Hywel-Jones & Spatafora, IMA Fungus 8: 349 (2017).

##### Description and illustration.

See Hywel-Jones (1996).

##### Host.

Spiders (Araneae, Thomisidae, *Amyciaea* sp.).

##### Habitat.

Specimens were found on the underside of dicot leaves and bamboo leaves of forest plants.

##### Material examined.

Thailand, Ranong Province, Khlong Nakha Wildlife Sanctuary, 9°27'34.52"N, 98°30'16.15"E, on spider (Araneae), 21 April 1994, Hywel-Jones NL, Nasit R, Plomhan R, Sivichai S, Thienhirun S, NHJ 3531 holotype, holotype damaged and no culture living, Neotype designated here: THAILAND, Ranong Province, Khlong Nakha Wildlife Sanctuary, 9°27'34.52"N, 98°30'16.15"E, on spider (Non-web builder, Araneae, Thomisidae, *Amyciaea* sp.), 21 April 1994, Hywel-Jones NL, Nasit R, Plomhan R, Sivichai S, Thienhirun S, NHJ 03510 (BBH 2649, holotype), ex-type culture BCC 6839.

**Figure 5.**
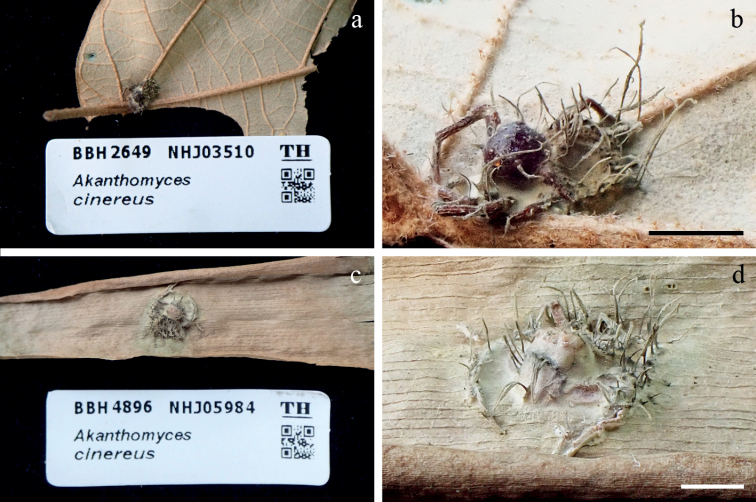
*Jenniferiacinerea***a, b** fungus on a spider (BBH 2649, NHJ 03510, BCC 6839) **c, d** fungus on a spider (BBH 4896, NHJ 05984, BCC 2191). Scale bars: 5 mm (**b, d**).

##### Notes.

**Figure 6.**
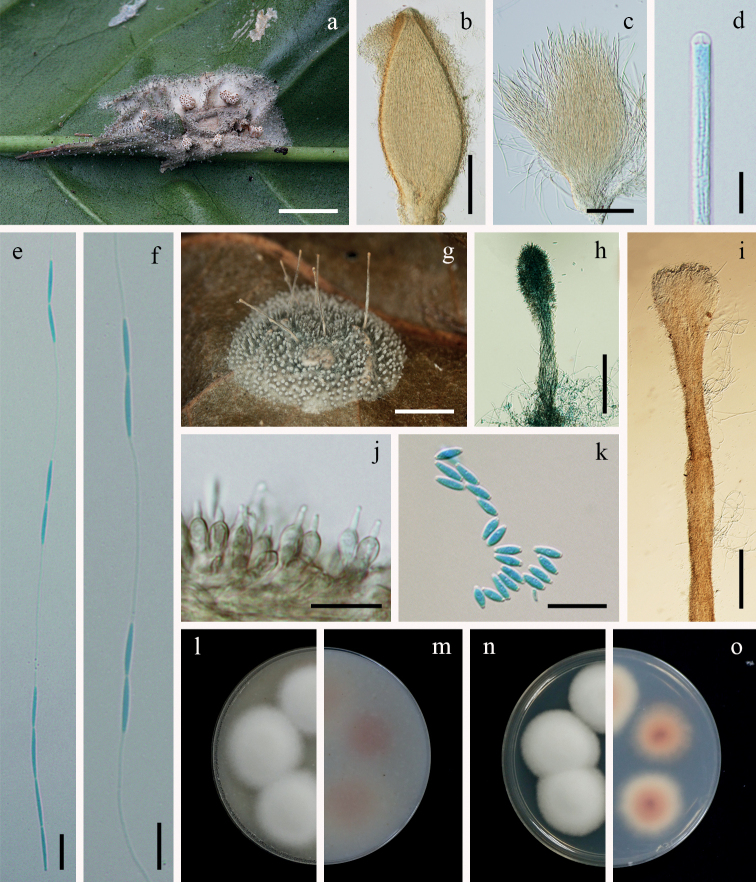
*Jenniferiagriseocinerea***a** fungus on a spider (BBH 29656) **b** perithecium **c** asci **d** ascus tip **e, f** whole ascospores with septate part-spores alternately connected with thread-like structures **g** fungus on a spider (BBH 33219) **h** short synnema **i** long synnema **j** phialides **k** conidia **l, m** colonies on OA at 21 days (**l** obverse, **m** reverse) **n, o** colonies on PDA at 21 days (**n** obverse, **o** reverse). Scale bars: 2 mm (**a, g**); 200 µm (**b**); 100 µm (**c, h, i**); 10 µm (**d, e, f, j, k**).

Based on the asexual morph of species in *Jenniferia*, they share similar characteristics in producing grey mycelium covering the spider host and multiple cylindrical synnemata from all parts of the host. The phylogenetic analysis supported *J.cinerea* as a sibling species to *J.griseocinerea*, but they have differences in producing synnemata. *Jenniferiacinerea* produces long synnemata, while *J.griseocinerea* produces short and long synnemata (Fig. 6). *Jenniferiacinerea* was not found as a sexual morph, whereas both *J.griseocinerea* and *J.thomisidarum* were found with sexual and asexual morphs (Tables 2 and 3). The shape of conidia in *J.cinerea* is clavate, but conidia in *J.griseocinerea* are fusiform and in *J.thomisidarum* are fusiform to cylindrical (Table 3). The spider hosts of *J.cinerea* from both specimens presented herein are identified as *Amyciaea* sp. belonging to the family Thomisidae.

#### ﻿ Jenniferia griseocinerea

Taxon classificationFungiHypocrealesCordycipitaceae

Tasanathai, Noisripoom & Mongkolsamrit sp. nov.

972B0606-DB8B-5815-92DA-78F28FC60841

 843091

Fig. 6

##### Typification.

Thailand, Nakhon Ratchasima Province, Khao Yai National Park, 14°26'20.72"N, 101°22'20.02"E, on spider (Non-web builder, Araneae, Thomisidae, *Diaea* sp.) attached to the underside of a dicot leaf of forest plants, 31 May 2010, K. Tasanathai, P. Srikitikulchai, S. Mongkolsamrit, T. Chohmee, A. Khonsanit, R. Somnuk, K. Sansatchanon, MY6006.01 (BBH 29656, holotype), ex-type culture BCC 42062 isolated from ascospores.

##### Etymology.

Named after the colour of the fresh specimens, from the Latin ‘*griseo*’, referring to dark grey and ‘*cinerea*’ meaning ash grey.

##### Description.

Spider hosts covered by yellowish-grey mycelium (156C). Sexual morph: *Stromata* non-stipitate. *Perithecia* growing in subiculum, aggregated in clusters, superficial, ovoid, 650–850 × 250–320 µm, ostiole pale brown. *Asci* cylindrical, 8-spored, 375–460 µm long, 5–6 µm broad, with cap 2–6 µm thick. *Ascospores* hyaline, whole ascospores with septate part-spores alternately connected with thread-like structures, four-terminal cells on each end with six alternating pairs of cells and filaments, sixteen cells per ascospore, up to 400 µm long, each cell narrowly fusiform, 10–15 × 1–2 µm, filiform regions, 35–45 × 0.2–0.8 µm. Asexual morph: Two types of synnemata were produced from all parts of the hosts. Several long synnemata, grey becoming pale brown at terminal ends, cylindrical with blunt end, 2.5–5 mm long, 100–150 μm broad, middle of long synnemata, 50–80 μm broad. *Conidiogenous cells* producing along long synnemata. Short synnemata, pale grey to dark grey, cylindrical, up to 450 µm long, 20–50 µm broad. *Conidiogenous cells* producing at the upper part of synnemata. *Phialides* flask-shaped at the base, 5–10 × 3–5 µm, tapering into distinct necks, 2–3.5 × 0.5–1 µm. *Conidia* hyaline, fusiform, 3–6 × 1–2 µm.

##### Culture characteristics.

Colonies on OA attaining a diam. of 18–20 mm in 21 days, cottony with high mycelium density, white, reverse pale yellow (165D). *Conidia* and reproductive structures not observed.

Colonies on PDA attaining a diam. of (16)17–20 mm in 21 days, cottony with high mycelium density, white, reverse pale yellow (165D). *Conidia* and reproductive structures not observed.

##### Host.

Spiders (Araneae, Thomisidae, Diaeacf.dorsata, *Diaea* sp.).

##### Habitat.

Specimens were found on the underside of dicot leaves of forest plants.

##### Additional materials examined.

Thailand, Nakhon Ratchasima Province, Khao Yai National Park, 14°26'20.72"N, 101°22'20.02"E, on spider (Non-web builder, Araneae, Thomisidae, *Diaea* sp.) attached to the underside of a dicot leaf of forest plants, 31 May 2010, K. Tasanathai, P. Srikitikulchai, S. Mongkolsamrit, T. Chohmee, A. Khonsanit, R. Somnuk, K. Sansatchanon, MY6006.02 (BBH 29656, paratype) ex-paratype culture BCC 42063 isolated from conidia; idem, on spider (Non-web builder, Araneae, Thomisidae, Diaeacf.dorsata) attached to the underside of a dicot leaf of forest plants, 8 November 2012, S. Mongkolsamrit, A. Khonsanit, W. Noisripoom, P. Srikitikulchai, R. Somnuk, MY8241 (BBH 33219) culture BCC 57821 isolated from conidia; idem, 9 August 2012, K. Tasanathai, S. Mongkolsamrit, A. Khonsanit, W. Noisripoom, K. Sansatchanon, MY7627 (BBH 36128) culture BCC 54893 isolated from conidia.

##### Notes.

Based on the multi-gene phylogenetic analyses presented in Fig. 1, *Jenniferiagriseocinerea* is closely related to *J.cinerea*. It shares similarity with *J.cinerea* in the production of several cylindrical synnemata arising from all parts of the spider host. However, *J.griseocinerea* differs from *J.cinerea* in producing long and short synnemata, while *J.cinerea* produces only long synnemata. The shape of phialides in *J.griseocinerea* from the specimens differs from *J.cinerea* and *J.thomisidarum*. Phialides in *J.griseocinerea* are flask-shaped, while phialides in *J.cinerea* and *J.thomisidarum* are cylindrical. Conidia in *J.griseocinerea* and *J.thomisidarum* are fusiform, occasionally cylindrical in *J.thomisidarum*. The conidia in *J.griseocinerea* are shorter than those reported for *J.thomisidarum* (3–6 × 1–2 µm vs. 3–12 × 1–3 µm) (Table 3).

#### ﻿ Jenniferia thomisidarum

Taxon classificationFungiHypocrealesCordycipitaceae

Mongkolsamrit, Noisripoom & Tasanathai sp. nov.

070A1D30-91F8-599B-AC8C-CF07E933BA18

 843092

Fig. 7

##### Typification.

Thailand, Nakhon Ratchasima Province, Khao Yai National Park, 14°26'20.72"N, 101°22'20.02"E, on spider (Non-web builder, Araneae, Thomisidae, Diaeacf.dorsata) attached to the underside of a dicot leaf of forest plants, 23 July 2009, K. Tasanathai, P. Srikitikulchai, S. Mongkolsamrit, R. Ridkaew, MY5032.01 (BBH 29502, holotype), ex-type culture BCC 37881 isolated from ascospores.

**Figure 7.**
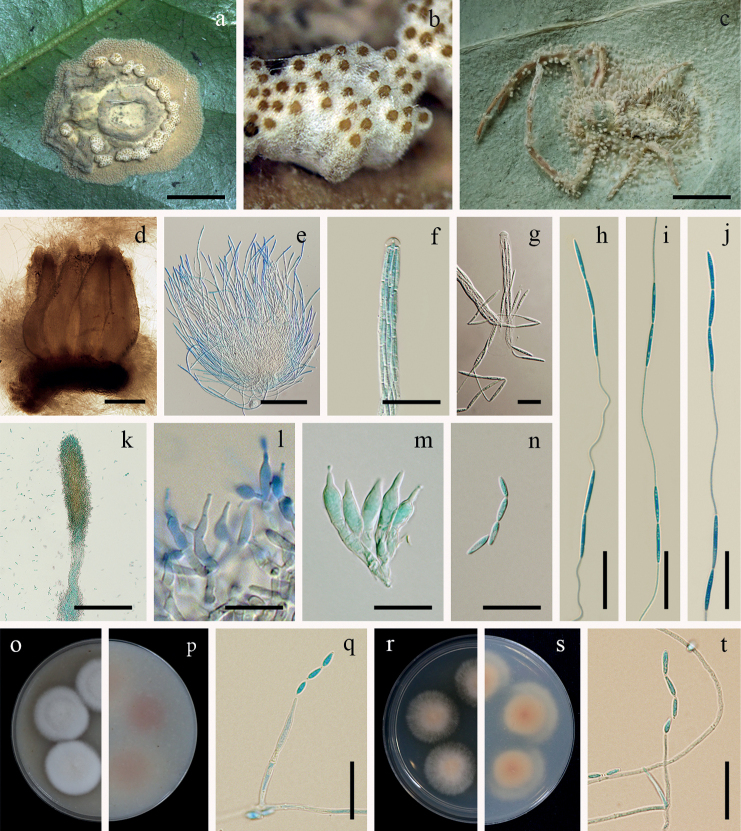
*Jenniferiathomisidarum***a** fungus on a spider (BBH 29502) **b** perithecia **c** fungus on a spider (BBH 30660) **d** perithecia **e** asci **f, g** ascus tip **h–j** whole ascospores with septate part-spores alternately connected with thread-like structures **k** synnema with conidiogenous cells **l, m** phialides **n** conidia **o**, **p** colonies on OA at 21 days (**o** obverse, **p** reverse) **q** phialide with conidia on OA**r, s** colonies on PDA at 21 days (**r** obverse, **s** reverse) **t** phialide with conidia on PDA. Scale bars: 2 mm (**a, c**); 300 µm (**d**); 200 µm (**e**); 100 µm (**k**); 10 µm (**f, g, l, m, n**); 20 µm (**h, i, j, q, t**).

##### Etymology.

Named after the host belonging to the family Thomisidae (Araneae).

##### Description.

Spider hosts covered by dense greyish-brown mycelium (199C–D). Sexual morph: *Stromata* non-stipitate. *Perithecia* growing in subiculum, aggregated in clusters, superficial, obpyriform, 850–1100 × 300–400 µm, ostiole pale brown. *Asci* cylindrical, 8-spored, 520–700 µm long, 4–6 µm broad, with cap 2–6 µm thick. *Ascospores* hyaline, whole ascospores with septate part-spores alternately connected with thread-like structures, three-terminal cells on each end with six alternating pairs of cells and filament, eighteen cells per ascospore, up to 680 µm long, each cell narrowly fusiform, 10–20 × 1–2 µm, filiform regions, 30–50 × 0.2–0.8 µm. Asexual morph: *Synnemata* arising from the mycelial mat, numerous, greyish-brown, cylindrical to clavate, erect up to 800 µm long, 30–100 µm broad. *Conidiogenous cells* producing at the upper part of synnemata, mostly monophialidic or some polyphialidic. *Phialides* cylindrical, (7)10–15(16) × 2–4(5) µm, tapering into a distinct neck, (1)1.5–3.5(5) × 1–1.5 µm. *Conidia* hyaline, fusiform, cylindrical, (3)8.5–10.5(12) × 1–3 µm.

##### Culture characteristics.

Colonies on OA attaining a diam. of (12)14–15 mm in 21 days, cottony with high mycelium density, white, reverse pale orange (165D), poor sporulation. *Phialides* arising from aerial hyphae, solitary, awl-shaped, lecanicillium-like, 20–40 × 1–2 µm. *Conidia* in chains, hyaline, fusiform, cylindrical, smooth, (3)7.5–10.5(12) × (1.5)2–2.5(3) µm.

Colonies on PDA attaining a diam. of 8–10 mm in 21 days, cottony with high mycelium density in the middle of colonies, mycelium with low density around the margin of colonies, pale orange, reverse moderate orange (167D), poor sporulation. *Phialides* arising from aerial hyphae, solitary, awl-shaped, lecanicillium-like, 10–35 × 1–2 µm. *Conidia* in the chains, hyaline, fusiform, cylindrical, smooth, (3)6.5–9.5(10) × (1.5)2–2.5(3) µm.

##### Host.

Spiders (Araneae, Thomisidae, Diaeacf.dorsata) .

##### Habitat.

Specimens were found on the underside of dicot leaves of forest plants.

##### Additional materials examined.

Thailand, Nakhon Ratchasima Province, Khao Yai National Park, 14°26'20.72"N, 101°22'20.02"E, on spider (Non-web builder, Araneae, Thomisidae, Diaeacf.dorsata) attached to the underside of a dicot leaf of forest plants, 23 July 2009, K. Tasanathai, P. Srikitikulchai, S. Mongkolsamrit, R. Ridkaew, MY5032.02 (BBH 29502, paratype), ex-paratype culture BCC 37882 isolated from conidia; idem, 7 August 2011, K. Tasanathai, P. Srikitikulchai, S. Mongkolsamrit, A. Khonsanit, W. Noisripoom, K. Sansatchanon, MY6813 (BBH 30660, culture BCC 48932); idem, 3 August 2011, K. Tasanathai, P. Srikitikulchai, S. Mongkolsamrit, A. Khonsanit, W. Noisripoom, K. Sansatchanon, MY6866 (BBH 30690), culture BCC 49257; idem, 9 August 2012, K. Tasanathai, S. Mongkolsamrit, A. Khonsanit, W. Noisripoom, MY7598 (BBH 32822), culture BCC 54482; MY7599 (BBH 32823), culture BCC 54483; MY7600 (BBH 32824), culture BCC 32824; idem, 26 June 2012, K. Tasanathai, P. Srikitikulchai, S. Mongkolsamrit, A. Khonsanit, W. Noisripoom, K. Sansatchanon, R. Somnuk, MY8636 (BBH 35789), culture BCC 64182; idem, 7 August 2013, P. Srikitikulchai, S. Mongkolsamrit, A. Khonsanit, W. Noisripoom, MY8878 (BBH 336396), culture BCC 66224.

**Notes.** In sexual morph specimens found in nature, *Jenniferiathomisidarum* resembles *J.griseocinerea* by the formation of non-stipitate ascomata. The perithecia of both species are superficial and aggregate in clusters, challenging the identification of the species rank. The ascospores are of the same type by septate part-spores alternately connected with thread-like structures along the whole ascospore (Fig. 2d). Ascospores in *J.thomisidarum* are longer than those reported for *J.griseocinerea* (Table 2). *Jenniferiathomisidarum* differs from *J.griseocinerea* in the size and shape of the perithecia and asci. In *J.thomisidarum*, perithecia and asci are larger and longer than those reported for *J.griseocinerea* (850–1100 × 300–400 µm vs. 650–850 × 250–320 µm; 520–700 × 4–6 µm vs. 375–460 × 5–6 µm). The perithecia in *J.thomisidarum* are obpyriform, while perithecia in *J.griseocinerea* are ovoid.

### ﻿Key to the species of *Jenniferia*

Based on sexual state characters

**Table:** 

1	Ascospores septate part-spores alternately connected with thread-like structures along the whole ascospore, non-stipitate ascomata, superficial perithecia up to 400 µm long	***J.griseocinerea***
–	Ascospores septate part-spores alternately connected with thread-like structures along the whole ascospore, non-stipitate ascomata, superficial perithecia to 680 µm long	***J.thomisidarum***

Based on asexual state characters

**Table:** 

1	Synnemata, multiple, two types of synnemata, long synnemata cylindrical with a blunt end, short synnemata	***J.griseocinerea***
–	Synnemata, multiple, one type of synnemata	**2**
2	Conidia 3.5–5.5 × 1–1.5 µm, clavate	***J.cinerea***
–	Conidia, 3–12 × 1–3 µm, fusiform, cylindrical	***J.thomisidarum***

#### ﻿ Parahevansia

Taxon classificationFungiHypocrealesCordycipitaceae

Mongkolsamrit & Noisripoom gen. nov.

F9747AA2-1142-5E66-9992-A11B85ED9B2F

 844040

##### Type species.

*Parahevansiakoratensis* (Hywel-Jones) Mongkolsamrit & Noisripoom, comb. nov., Mycol. Res. 100: 1067 (1996).

##### Etymology.

Morphologically resembling the genus *Hevansia*, but being phylogenetically distinct.

##### Description.

Asexual morph: *Synnemata* arising from all parts of host, numerous, simple, brown at the sterile base becoming grey white with fertile part. *Conidiogenous cells* phialidic producing upper part of the synnemata. *Phialides* in a monolayer, single on basal lateral cells of synnemata, crowded, obovoid to ellipsoid with distinct necks. *Conidia* in chain, hyaline, smooth-walled, clavate.

##### Notes.

*Parahevansiakoratensis*, the type species of this genus, was originally described as species of *Akanthomyces* (Hywel-Jones, 1996) and later transferred to *Hevansia* (Kepler et al. 2017). Our multi-gene phylogenetic analyses supported *Parahevansia* as a monophyletic clade with strong support (MLB = 100 / BPP = 1.00, Fig. 1). Therefore, we introduced *Parahevansia* as a new genus that accommodates a single species, *Pa.koratensis*.

#### ﻿ Parahevansia koratensis

Taxon classificationFungiHypocrealesCordycipitaceae

(Hywel-Jones) Mongkolsamrit & Noisripoom, comb. nov.,

FCB48883-0BD3-50D6-BDAA-22316B1C4B9B

 844041

≡ Akanthomyceskoratensis Hywel-Jones, Mycol. Res. 100: 1068 (1996).
≡ Hevansiakoratensis (Hywel-Jones) Luangsa-ard, Hywel-Jones & Spatafora, IMA Fungus 8: 349 (2017).

##### Typification.

Thailand, Nakhon Ratchasima Province, Khao Yai National Park, 14°26'20.72"N, 101°22'20.02"E, on spider (Araneae, Salticidae), 12 December 1991, N.L. Hywel-Jones, NHJ 666.01 holotype.

##### Description and illustration.

See Hywel-Jones (1996).

##### Host.

Spider (Araneae, Salticidae).

##### Habitat.

Specimens were found on the underside of dicot leaves of forest plants.

##### Notes.

Both *Parahevansiakoratensis* and *H.novoguineensis* occur on spiders and both produce white mycelium with reddish pigment diffusing in agar media (Hywel-Jones 1996). However, the sporulation of *H.novoguineensis* is produced on media, while no sporulation on media in *Pa.koratensis* was observed. Based on the phylogenetic tree (Fig. 1), NHJ 2662 clustered with the ex-type strain NHJ 666.01 of *Pa.koratensis*. The insect host of the strain NHJ 2662 was recorded as a Lepidoptera larva. This result shows that *Pa.koratensis* is parasitic on spiders and Lepidoptera larva.

#### ﻿ Polystromomyces

Taxon classificationFungiHypocrealesCordycipitaceae

Mongkolsamrit, Noisripoom, Sakolrak & Himaman gen. nov.

9A09E9CA-2810-5CD3-BC01-3DD610D02B1B

 843093

##### Type species.

*Polystromomycesaraneae* Mongkolsamrit, Noisripoom, Sakolrak & Himaman.

##### Etymology.

From Latin “poly” (many), referring to many stromata of the fungus on the host.

##### Description.

Sexual morph: *Stromata* stipitate, multiple, pale yellow mycelium covering the host. Stipes arising from spider egg sac, cylindrical at the base, slightly enlarged midway to the terminal end of the stipe below the fertile head. *Fertile heads* produce at the terminal stipes, disc-shaped, upper surface slightly convex. *Perithecia* completely immersed, ovoid. *Asci* cylindrical. *Ascospores* hyaline, filiform, disarticulating into part-spores. Colony on PDA and OA, white, producing microcycle conidiation.

##### Notes.

*Polystromomyces* contains a new species, *Po.araneae*. It shares similarity with species in *Hevansia* in producing multiple stipes with fertile heads at the apex. This specimen is found on a spider egg sac (Araneae) attached to the underside of a dicot leaf. There is no record of the asexual morph on the specimen.

#### ﻿ Polystromomyces araneae

Taxon classificationFungiHypocrealesCordycipitaceae

Mongkolsamrit, Noisripoom, Sakolrak & Himaman sp. nov.

664FD2BB-9690-5008-B84D-3AC9DC881EE1

 843094

Fig. 8

##### Typification.

Thailand, Tak Province, Umphang Wildlife Sanctuary, 15°55'36.33"N, 98°45'12.15"E, on spider egg sac (Araneidae*sensu lato*) attached to the underside of a dicot leaf, 6 December 2020, B. Sakolrak, MY12684 (BBH 49054, holotype), ex-type culture BCC 93301 isolated from ascospores.

**Figure 8.**
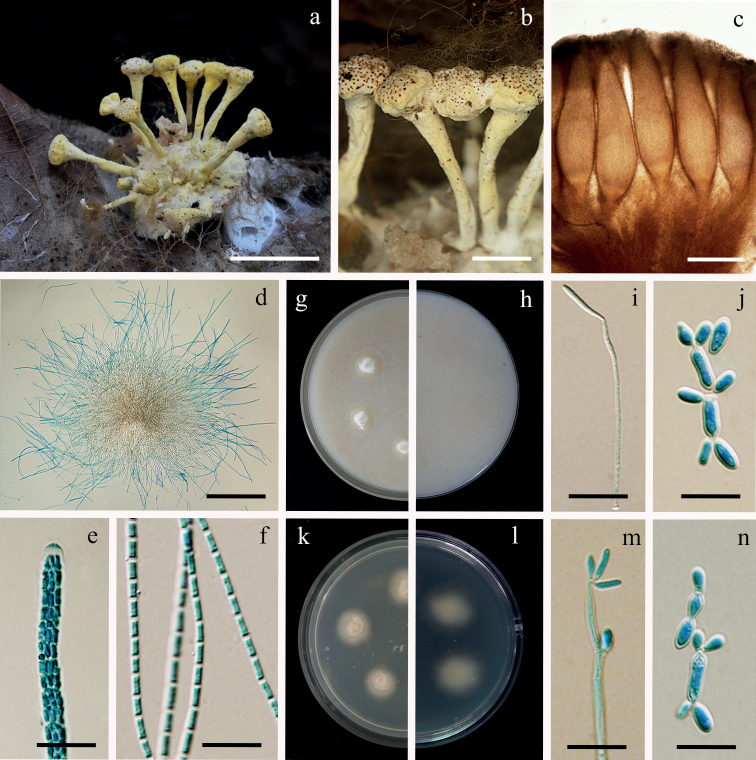
*Polystromomycesaraneae***a** fungus on a spider egg sac (BBH 49054) **b** fertile heads **c** perithecia **d** asci **e** ascus tip **f** part-spores **g, h** colonies on OA at 21 days (**g** obverse, **h** reverse) **i** conidium formation from a hypha on OA**j** microcycle conidiation on OA**k, l** colonies on PDA at 21 days (**k** obverse, **l** reverse) **m** conidia formation from a hypha on PDA **n** microcycle conidiation on PDA. Scale bars: 10 mm (**a**); 3 mm (**b**); 200 µm (**c, d**); 10 µm (**e, f, i, j, m, n**).

##### Etymology.

From Latin, “aranea” refers to a spider host.

##### Description.

Hosts covered by dense pale yellow mycelium (162D). Sexual morph: *Stromata* stipitate, arising from the host, multiple, cylindrical at the base, slightly enlarged midway to the terminal stipe below the fertile head, moderate yellow (162A–B), 8–12 mm long, 1–3 mm broad. *Fertile head* disc-shaped, upper surface slightly convex, 3–4 × 2–3.5 mm. *Perithecia* completely immersed, narrowly ovoid, 1000–1400 × 200–350 µm, ostiole pale brownish-orange (165B). *Asci* cylindrical, 8-spored, 400–1000 µm long, 3.5–6 µm broad, with cap 2–5 µm thick. *Ascospores* hyaline, dissociating into 128 part-spores, cylindrical, 2–6 × 1–3 µm.

##### Culture characteristics.

Colonies on OA attaining a diam. of 8–10 mm in 21 d, mycelium sparse, white, reverse pale yellow (161C). *Conidia* forming on vegetative hyphae or by microcyclic conidiation, hyaline, clavate to cylindrical, 2–10 × 1–5 µm.

Colonies on PDA attaining a diam. of 8–10 mm in 20 d, mycelium sparse, white, reverse pale yellow (161C). *Conidia* forming on vegetative hyphae or by microcyclic conidiation, hyaline, clavate to cylindrical, 2–12 × 1–5 µm.

##### Host.

Spider egg sac.

##### Habitat.

Specimen was found on the underside of a dicot leaf of a forest plant.

##### Notes.

Based on natural specimens, *Po.araneae* closely resembles *H.nelumboides* and *H.novoguineensis* by producing fertile heads at the end of the stipes. The perithecia of these species are completely immersed. The ascospores of *Po.araneae* and *H.nelumboides* are filamentous, multiseptate ascospores disarticulating into part-spores, whereas *H.novoguineensis* produces filiform, whole ascospores. However, *Po.araneae* differs from *H.nelumboides* in the size of the perithecia. In *Po.araneae*, perithecia are larger than those reported for *H.nelumboides* (1000–1400 × 200–350 µm vs. 535–545 × 180–190 µm) (Table 2). *Polystromomycesaraneae* produces microcycle conidiation from conidia on culture, while the microcyclic sporulation is often seen in discharged ascospores in *Metarhiziumphuwiangense* Luangsa-ard, Mongkols., Himaman, Thanakitp. & Samson and *Purpureomyceskhaoyaiensis* (Hywel-Jones) Luangsa-ard, Samson & Thanakitp (Mongkolsamrit et al. 2020a).

## ﻿Discussion

In this study, we conducted comparative morphological studies and phylogenetic analyses of spider parasitic fungi belonging to *Hevansia*, *Jenniferia*, *Parahevansia* and *Polystromomyces*. Kepler et al. (2017) established *Hevansia* with two species, i.e. *H.nelumboides* and *H.novoguineensis*, based on a split inferred from molecular data. Our molecular analyses revealed the sexual-asexual link between the Thai material (BCC 42675) and the ex-type culture of *H.novoguineensis* (CBS 610.80) and a novel species, *H.minuta* (Fig. 1). The sexual morph morphological characters in *Hevansia* (observed in *H.novoguineensis*, *H.minuta* and *H.nelumboides*) include stipes with terminal fertile heads arising from the dorsal region of their spider hosts (Figs 3a and 4a, this study; Fig. 3J in Kepler et al. 2017). Hevansiacf.novoguineensis (BCC 2093 and NHJ 4314) formed a subclade genetically close to *H.novoguineensis*, but the herbarium materials of these strains were not available for comparison. Considering that H.cf.novoguineensis formed a sister clade to *H.novoguineensis* (Fig. 1), but this relation was not consistently found between the markers, we propose that *H.novoguineensis* is a species complex and that H.cf.novoguineensis could potentially be considered as a different species if more molecular markers could unambiguously demonstrate its separation from the clade containing the ex-type strain.

In this study, the genus *Polystromomyces* is established with a single species (*Po.araneae*); it formed the basal lineage to *Hevansia*, *Jenniferia* and *Gibellula* and shared the same ecological habitat (on the underside of dicot leaves of forest plants). *Polystromomycesaraneae* shares morphological similarity to *Hevansia* by producing multiple stromata with fertile heads at the terminal part of stipes. Notably, *Po.araneae* can be distinguished from *Hevansia* by the shape of stipes. The stipes in *Polystromomyces* are cylindrical at the base and slightly enlarged midway to the terminal below the disc-shaped fertile heads. In contrast, the stipes of *Hevansia* are connected in a cylindrical arrangement with the fertile heads, resembling lotus seed pods on stems.

The novel genus *Jenniferia* was proposed to accommodate *Jenniferiacinerea*, *J.griseocinerea* and *J.thomisidarum*. Based on the natural specimens, the sexual morph of species within *Jenniferia* produce non-stipitate ascomata. The lack of stipe is a shared trait amongst pathogenic fungi species on spiders in Cordycipitaceae, such as *Gibellula* spp., *Akanthomycesthailandicus* and *A.sulphureus*, forming a torrubiella-like sexual morph (Mongkolsamrit et al. 2018; Kuephadungphan et al. 2020). However, species in *Jenniferia* described here can be easily distinguished from species in *Gibellula* spp., *A.thailandicus* and *A.sulphureus* by the superficial and aggregated perithecia in clusters forming a cushion (a distinctive character of *Jenniferia*), causing species in this genus to be easily recognisable in the field.

We reviewed valid species according to a current classification through molecular data combined with the observation of ascospore micro-morphology. Many studies revealed that cordycipitaceous fungi produced three types of ascospore morphology shown through the illustration and description in Figs 1 and 2(a–c). The filiform whole ascospores type (Fig. 2a) with the shape of thread is observed in *Akanthomycessulphureus*, *Blackwellomyces* spp., *Cordycepskuiburiensis*, *Hyperdermium* (e.g. *H.bertonii*, *H.pulvinatum*) and *Neotorrubiellachinghridicola* (Mongkolsamrit et al. 2018, 2020b; Crous et al. 2019; Sullivan et al. 2000; Thanakitpipattana et al. 2020). The presence of multiseptate ascospores disarticulating into part-spores (Fig. 2b) can be seen in several genera, such as *Akanthomyces* (e.g. *A.thailandicus*, *A.pyralidarum* and *A.noctuidarum*), *Beauveria* (e.g. *B.asiatica*, *B.gryllotalpidicola*), *Cordyceps* (e.g. *C.militaris*, *C.inthanonensis* and *C.nidus*) and also includes species in *Gibellula* (Mains 1958; Chiriví et al. 2017; Mongkolsamrit et al. 2018, 2020b; Aini et al. 2020; Kuephadungphan et al. 2020). The bola-ascospores morphology was noted in the description of *Cordycepsbifusispora* O.E. Erikss. and *Cordycepsninchukispora* (C.H. Su & H.H. Wang) G.H. Sung, J.M. Sung, Hywel-Jones & Spatafora (Fig. 2c) by Eriksson (1982) and Su and Wang (1986), respectively. Many *Cordyceps* species producing bola-ascospores were reported from Thailand and China (Tasanathai et al. 2016; Mongkolsamrit et al. 2018, 2020b; Wang et al. 2020). *Samsoniella*, a recent established genus also produces bola-ascospores (Mongkolsamrit et al. 2018; Wang et al. 2020). Examination of our specimens of *Jenniferia* revealed that its ascospores possess a unique shape not seen before in Cordycipitaceae. In this study, we are introducing another ascospore morphology (Fig. 2d), which is an autapomorphic character within *Jenniferia* that can be used to identify at the genus level.

There are two types of phialides in species of *Hevansia*. Some species produce globose to subglobose phialides with a distinct neck along the synnemata (e. g. *H.arachnophila*, *H.minuta*, *H.novoguineensis* and *H.ovalongata*), whereas other species produce phialides on the basal cells along the synnemata (e.g. *H.longispora* and *H.websteri*). These characters can be informative for recognising species of *Hevansia*. All species in *Jenniferia* produce the asexual morph and only two species are occasionally found producing sexual and asexual morphs on the same specimens, i.e. *J.griseocinerea* and *J.thomisidarum*. The *Jenniferia* asexual morph in nature differs from species in *Hevansia* in possessing pale grey to ash grey synnemata scattered over the body and legs of its host. Notably, *J.griseocinerea* significantly differs by producing two types of synnemata (Fig. 6g–i). In contrast, the anamorph of *Hevansia* (e.g. *H.novoguineensis*) produces white synnemata arising from the host (Fig. 3f).

Spider hosts associated with the *Jenniferia* species were identified as Diaeacf.dorsata for all specimens of *J.griseocinerea* and *J.thomisidarum*, except one specimen of *J.griseocinerea* that was identified as *Diaea* sp. Meanwhile, *Amyciaea* sp. is found as the host of *J.cinerea*. *Jenniferia* is, thus, up to now exclusively associated with the spider genera *Diaea* and *Amyciaea* in the family Thomisidae. A review by Shrestha et al. (2019) reported pathogenic fungi on spiders found in Thomisidae and includes *Gibellula* spp. on *Tmarus* spp. (Costa 2014), *Torrubiellaalbolanata* on a thomisid spider (Petch 1944) and *T.neofusiformis* on a thomisid spider (Kobayasi and Shimizu 1982). Recently, an additional species occurring on Thomisidae was found, including *Gibellulacebrennini* associated with Cebrenninuscf.magnus (Kuephadungphan et al. 2020).

*Hevansia* species are specialised parasites on spiders. *Parahevansia*, proposed as a new genus that accommodates *Pa.koratensis* (≡ *Akanthomyceskoratensis*), is parasitic on a salticid spider (Salticidae) and Lepidoptera larva (Hywel-Jones 1996; Shrestha et al 2019, in this study). *Polystromomycesaraneae* occurs on the spider egg sac (Araneidae*sensu lato*) attached to the underside of a dicot leaf. *Cordycepsaraneae* Mongkols., Tasan., Noisrip., Himaman & Luangsa-ard has also been reported on spider egg sac inhabiting the leaf litter (Mongkolsamrit et al. 2020b). Although *Po.araneae* is most similar to *H.nelumboides* and *H.novoguineensis* by producing stipes with fertile heads at the terminal, the two latter species are found on adult spiders.

## Supplementary Material

XML Treatment for Hevansia

XML Treatment for Hevansia novoguineensis

XML Treatment for Hevansia arachnophila

XML Treatment for Hevansia longispora

XML Treatment for Hevansia nelumboides

XML Treatment for Hevansia ovalongata

XML Treatment for Hevansia websteri

XML Treatment for Hevansia minuta

XML Treatment for Jenniferia

XML Treatment for Jenniferia cinerea

XML Treatment for Jenniferia griseocinerea

XML Treatment for Jenniferia thomisidarum

XML Treatment for Parahevansia

XML Treatment for Parahevansia koratensis

XML Treatment for Polystromomyces

XML Treatment for Polystromomyces araneae

## Supplementary materials

Supplementary material 1 Figures S1–S5 Data type Pdf file Explanation note RAxML trees of *Hevansia*, *Jenniferia*, *Parahevansia*, *Polystromomyces* and related genera in the Cordycipitaceae from different molecular markers. https://binary.pensoft.net/file/719482Copyright notice: This dataset is made available under the Open Database License (http://opendatacommons.org/licenses/odbl/1.0/). The Open Database License (ODbL) is a license agreement intended to allow users to freely share, modify, and use this Dataset while maintaining this same freedom for others, provided that the original source and author(s) are credited. Suchada Mongkolsamrit, Wasana Noisripoom, Kanoksri Tasanathai, Noppol Kobmoo, Donnaya Thanakitpipattana, Artit Khonsanit, Booppa Petcharad, Baramee Sakolrak, Winanda Himaman
