# Supplementary material for: ﻿Comprehensive treatise of Hevansia and three new genera Jenniferia, Parahevansia and Polystromomyces on spiders in Cordycipitaceae from Thailand
Source: MycoKeys. 2022 Jul 26;91:113–49. doi: 10.3897/mycokeys.91.83091 (PMC9849099; doi:10.3897/mycokeys.91.83091)
Supplement: Supplementary material 1 — Figures S1–S5 [file mycokeys-91-113-s001.pdf]

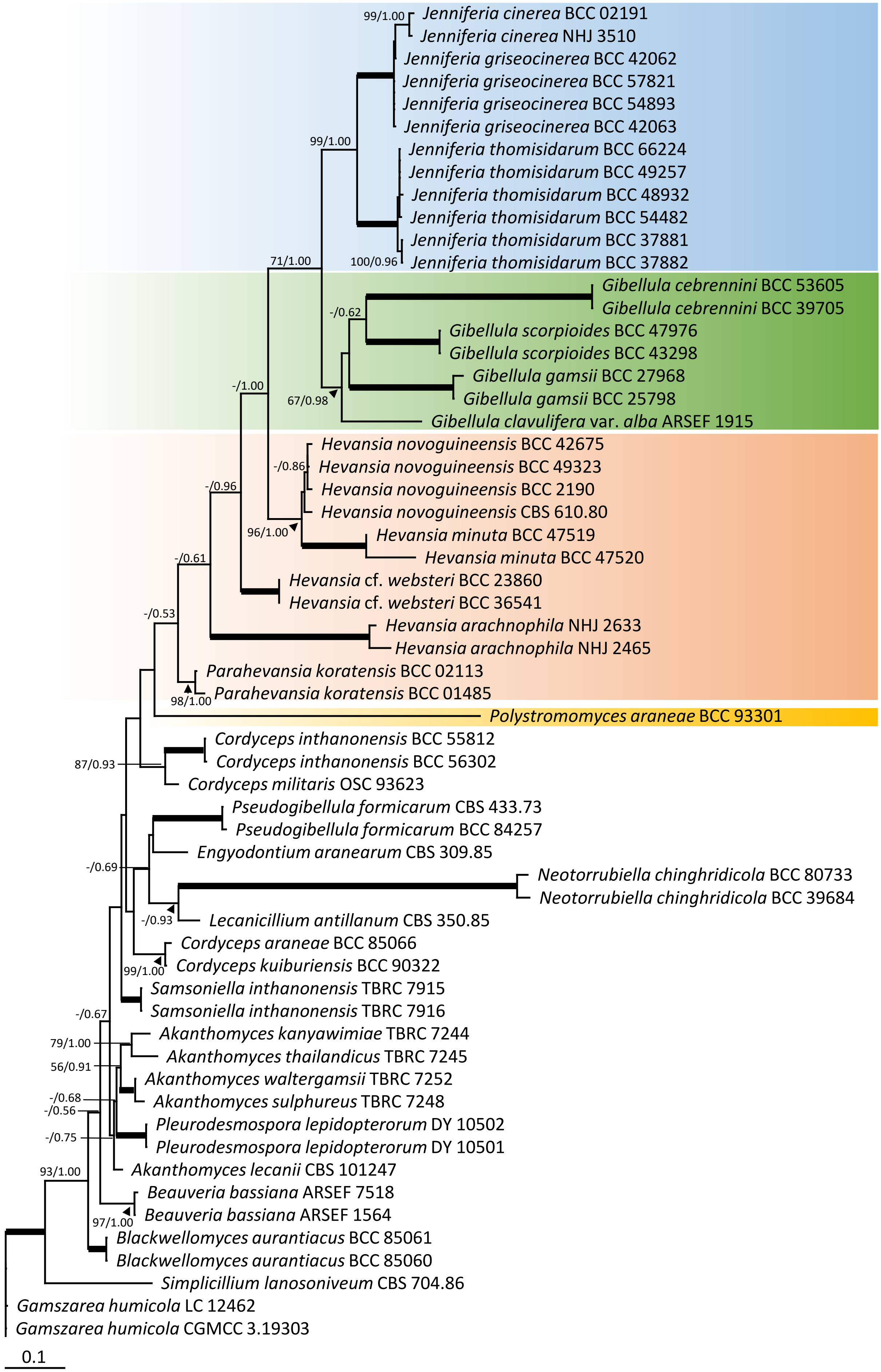

Suppl. material 1: Fig.1 RAxML tree of *Hevansia*, *Jenniferia*, *Parahevansia*, *Polystromomyces*, and related genera in the Cordycipitaceae from ITS dataset. Numbers at the major nodes represent maximum likelihood bootstrap (MLB) and Bayesian posterior probabilities (BPP). Bold lines in the tree represent 100% of MLB and 1.0 of BPP.

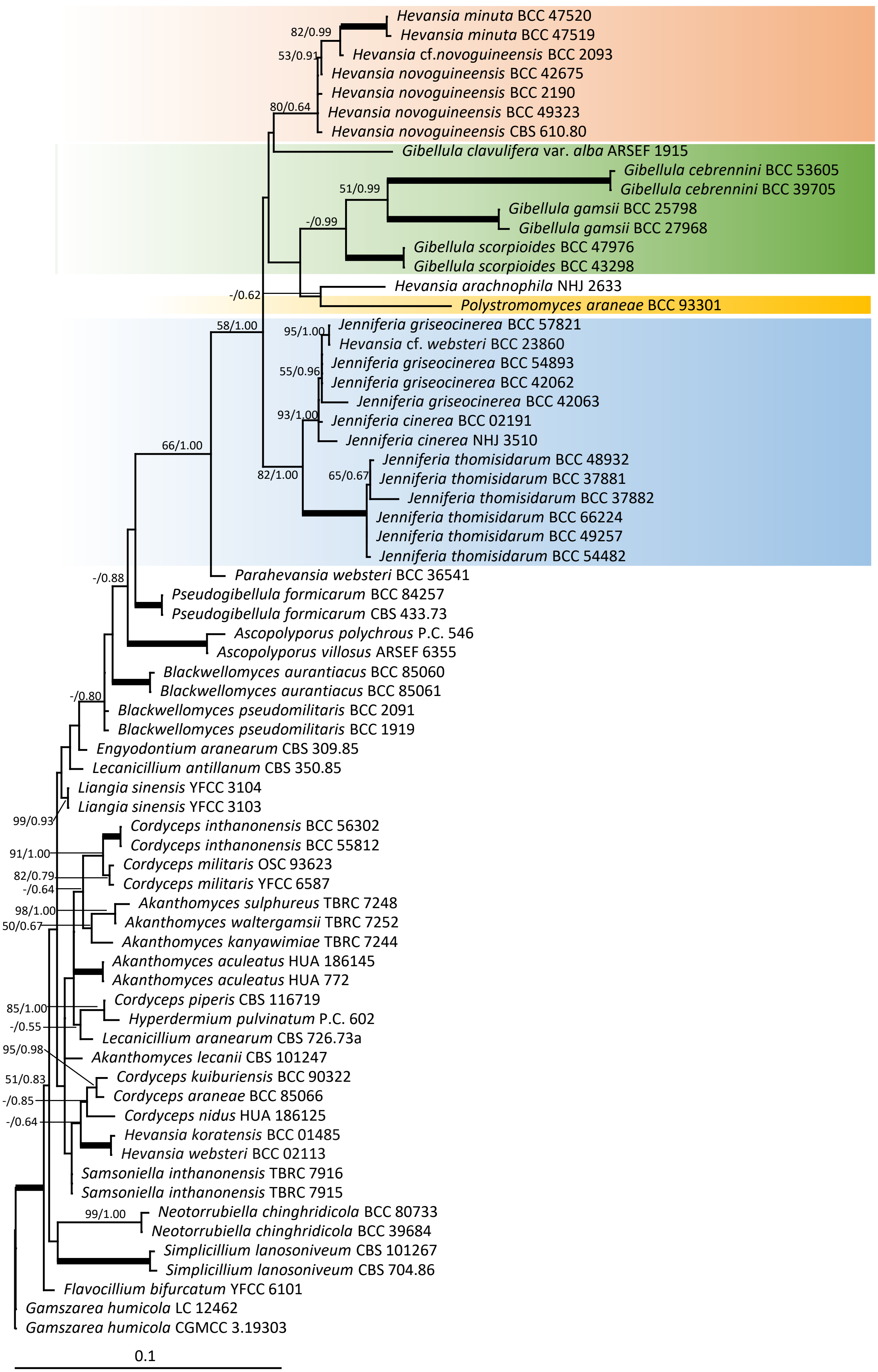

Suppl. material 1: Fig. 2 RAxML tree of *Hevansia*, *Jenniferia*, *Parahevansia*, *Polystromomyces*, and related genera in the Cordycipitaceae from LSU dataset. Numbers at the major nodes represent maximum likelihood bootstrap (MLB) and Bayesian posterior probabilities (BPP). Bold lines in the tree represent 100% of MLB and 1.0 of BPP.

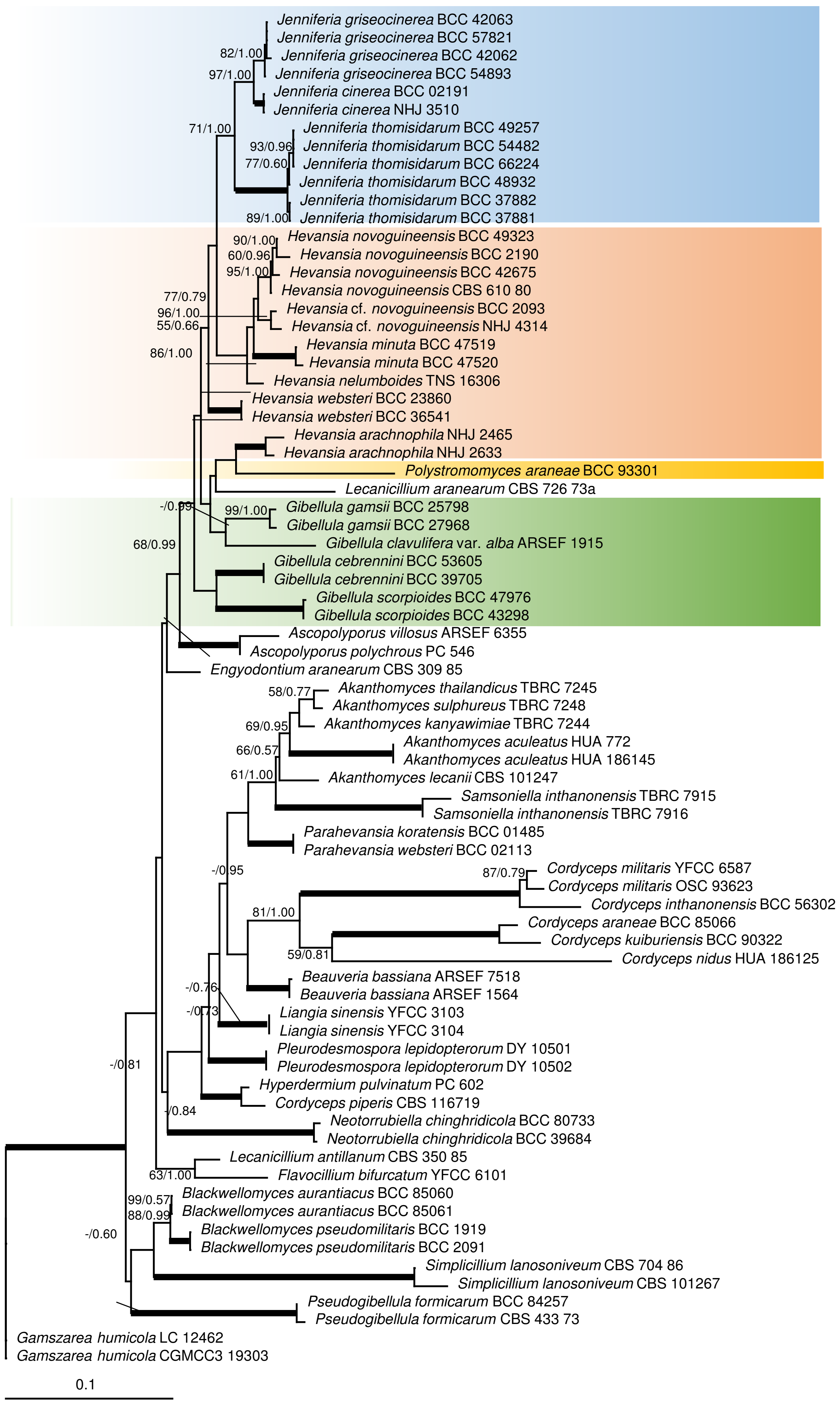

Suppl. material 1: Fig.3 RAxML tree of *Hevansia*, *Jenniferia*, *Parahevansia*, *Polystromomyces*, and related genera in the Cordycipitaceae from *tef1* dataset. Numbers at the major nodes represent maximum likelihood bootstrap (MLB) and Bayesian posterior probabilities (BPP). Bold lines in the tree represent 100% of MLB and 1.0 of BPP.

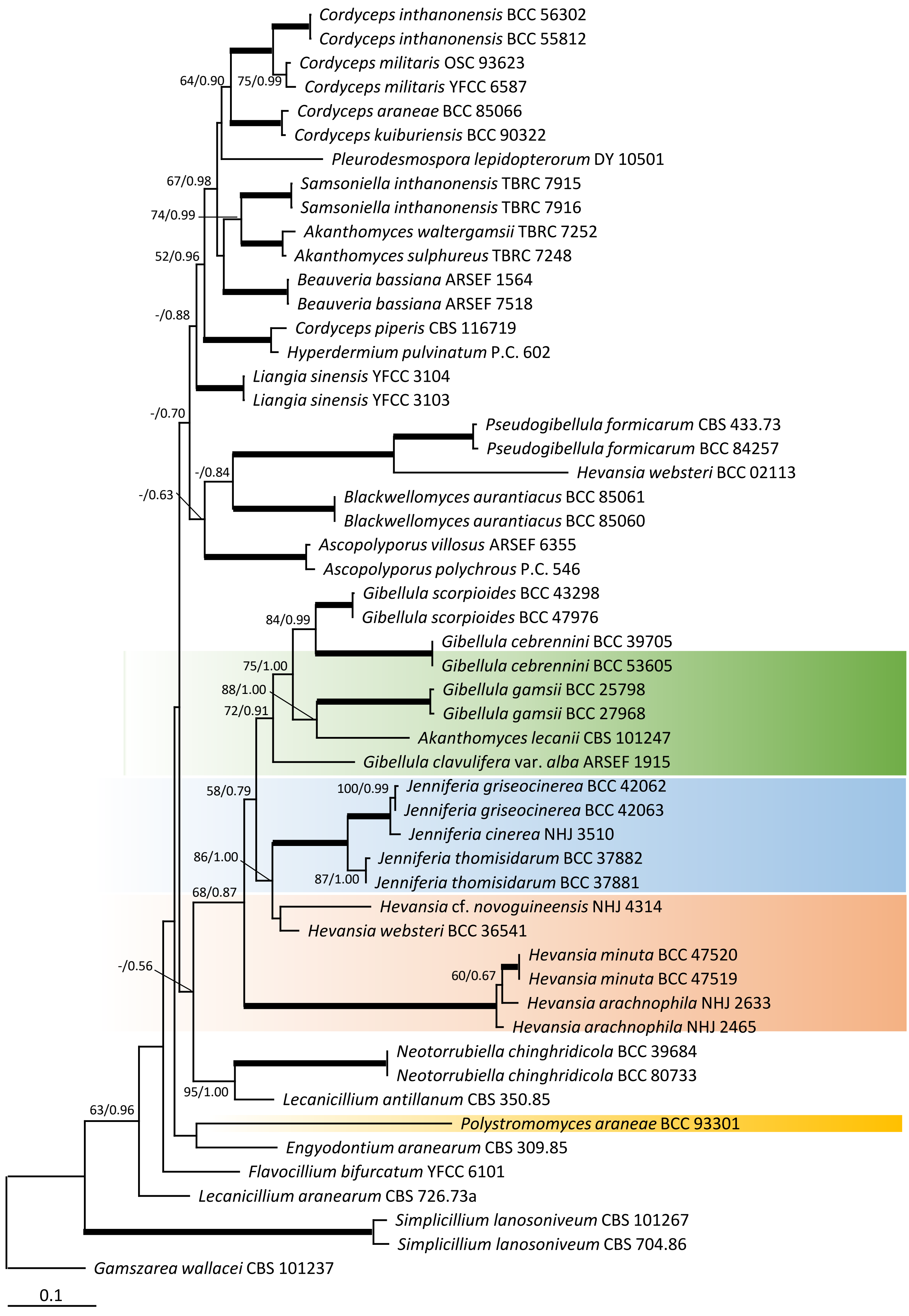

Suppl. material 1: Fig.4 RAxML tree of *Hevansia*, *Jenniferia*, *Parahevansia*, *Polystromomyces*, and related genera in the Cordycipitaceae from *rpb1* dataset. Numbers at the major nodes represent maximum likelihood bootstrap (MLB) and Bayesian posterior probabilities (BPP). Bold lines in the tree represent 100% of MLB and 1.0 of BPP.

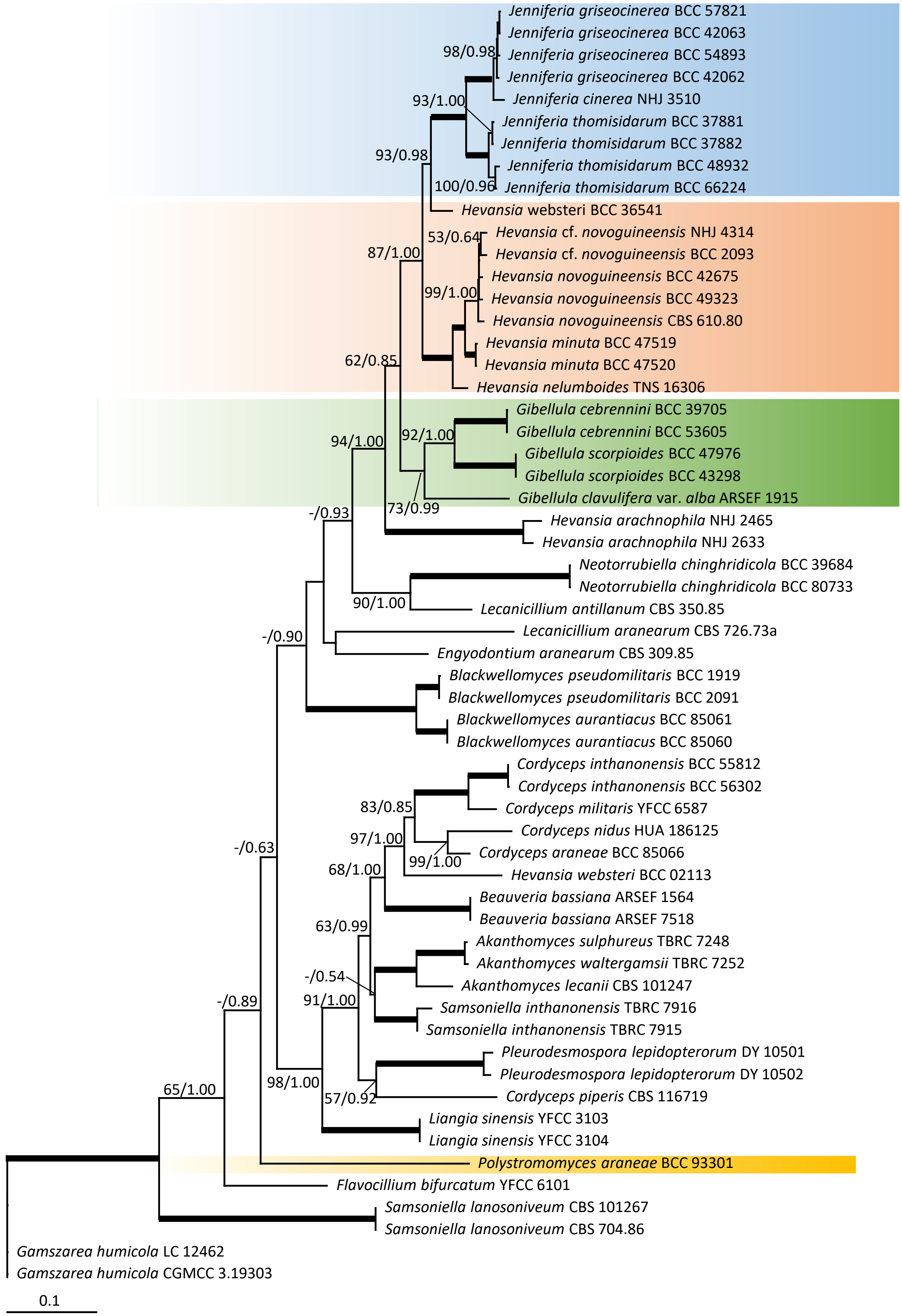

Suppl. material 5: Fig.1 RAxML tree of *Hevansia*, *Jenniferia*, *Parahevansia*, *Polystromomyces*, and related genera in the Cordycipitaceae from *rpb2* dataset. Numbers at the major nodes represent maximum likelihood bootstrap (MLB) and Bayesian posterior probabilities (BPP). Bold lines in the tree represent 100% of MLB and 1.0 of BPP.
